# A Magneto-Electric Device for Fluid Pipelines with Vibration Damping and Vibration Energy Harvesting

**DOI:** 10.3390/s24165334

**Published:** 2024-08-17

**Authors:** Yi-Ren Wang, Po-Chuan Huang

**Affiliations:** Department of Aerospace Engineering, Tamkang University, Tamsui District, NewTaipei City 25137, Taiwan; 612430032@o365.tku.edu.tw

**Keywords:** vibration energy harvesting system, fluid–structure interaction, method of multiple scales, piezoelectric energy conversion, magnetic dipole–dipole interactions (MDDIs)

## Abstract

This study introduces an innovative energy harvesting system designed for industrial applications such as fluid pipelines, air conditioning ducts, sewer systems, and subsea oil pipelines. The system integrates magneto-electric flow coupling and utilizes a dynamic vibration absorber (DVA) to mitigate the vibrations induced by fluid flow while simultaneously harvesting energy through magnetic dipole–dipole interactions in a vibration energy harvester (VEH). The theoretical models, based on Hamilton’s Principle and the Biot–Savart Law, were validated through comprehensive experiments. The results indicate the superior performance of the small-magnet system over the large-magnet system in both damping and power generation. The study analyzed the frequency response and energy conversion efficiency across different parameters, including the DVA mass, spring constant, and placement location. The experimental findings demonstrated significant vibration reduction and increased voltage output, validating the theoretical model. This research offers new avenues for energy harvesting systems in pipeline infrastructures, potentially enhancing energy efficiency and structural integrity.

## 1. Introduction

The global push towards green energy to mitigate global warming has spurred researchers to seek innovative methods for harnessing sustainable energy sources. Vibration energy harvesting, which involves capturing energy from structural vibrations, has garnered significant attention. The common sources of vibrational energy include bridges, roads, and railway tracks. However, pipeline vibrations, prevalent in many industrial facilities with extensive networks of gas and fluid pipelines, also hold substantial potential. Harvesting this untapped energy through vibration energy harvesting systems (VEHSs) can significantly contribute to meeting the renewable energy demands. This approach aligns with sustainable practices, emphasizing energy collection and reuse, which are crucial for the future.

In the realm of vibration energy harvesting (VEH) systems, Jia [1] conducted a comprehensive comparison of eight nonlinear vibration energy harvesters, highlighting their strengths and weaknesses. Notably, the conventional linear vibration energy harvesters are only suitable for fixed single-frequency environments and exhibit low mechanical energy accumulation efficiency. In contrast, nonlinear vibration energy harvesters offer distinct advantages, including broadened frequency bandwidth, enhanced power density, and increased responsiveness to noise and shock wave impacts. Lan et al. [2] emphasized the importance of maintaining a state of high-energy orbital oscillation in nonlinear vibration energy harvesters to achieve high-performance and wide-bandwidth energy harvesting. Researchers like Yang [3] argued that vibration energy harvesting systems find extensive applications in aviation, space exploration, machinery, sustainable energy engineering, and biomedicine. Beyond energy harvesting, these systems also play a crucial role in reducing harmful vibrations, offering protection, and prolonging the service life of instruments and equipment. Studies show that electromagnetic and piezoelectric vibration energy conversion mechanisms are widely accepted in engineering due to their simple design, ease of assembly, and high conversion efficiency.

While vibration energy harvesting systems (VEHSz) require the generation of substantial vibration energy, managing this energy without causing detrimental effects on the main structure and reducing its service life poses a significant challenge. To address this issue, the implementation of shock absorbers on the main body is both necessary and beneficial. Cadiou et al. [4] proposed that the impact of vibration could be effectively mitigated through the incorporation of tuned mass damper (TMD) components. However, adjusting the natural frequency of the linear TMD to match that of the main structure is crucial to prevent efficiency conversion losses. Recognizing the limitations associated with linear TMD, Cadiou et al. chose the nonlinear energy sink (NES) as the primary shock absorber system in their experiments. Despite its potential, the difficulty in controlling and identifying the damping coefficient during the design process hinders the full utilization of the analytical model described. Parseh et al. [5] compared TMD and NES systems, highlighting that the performance of an NES is contingent on the designed external force amplitude. An NES proves to be more effective for lower-external-force amplitudes, while a TMD outperforms it in scenarios with excessive amplitudes. In a distinct approach, Jin et al. [6] employed a tuned particle impact damper (TPID) to reduce the vibrations on rails and minimize the rolling noise. The damper design, integrating the features of a dynamic vibration absorber (DVA) and an impact damper, demonstrates exceptional vibration reduction capabilities at specific frequencies. Gupta et al. [7] utilized a Stockbridge damper (SD) to alleviate the eddy current chatter and prevent fatigue failure in the transmission lines. 

In addition to exploring dampers, the research in general vibration energy harvesting systems (VEHs) also delves into the realm of nonlinear beams. Forsat [8] introduced a high-order shear deformation beam theory for constructing hyperelastic beams composed of silicone rubber and unfilled natural rubber. Employing modular and nonlinear vibration analysis, they applied Hamilton’s Principle to derive the nonlinear equations and ascertain the nonlinear vibration frequencies. The results revealed that increasing amplitude correlates with higher frequencies, influenced by the nonlinear hardening effect. Liu et al. [9] developed an equivalent nonlinear beam model (ENBM) for a nonlinear beamlike truss (NBT) in force vibration analysis. This model utilized von Kármán nonlinear strains to introduce geometric nonlinearity, simulating the nonlinear behavior of the NBT. However, despite the dominant role of the first mode in the ENBM’s nonlinear response, under specific external excitation frequencies, the emergence of nonlinear resonance in the third mode highlights the significance of analyzing internal resonance—a phenomenon beyond the reach of the first mode. To address the complexity of nonlinear beam vibrations, Jing et al. [10] employed the extended Rayleigh–Ritz method. By incorporating time changes and multiple scales, they generated a first-order approximate nonlinear beam vibration equation. Utilizing resonance frequency, Euler–Bernoulli beam theory, and Timoshenko beam theory, their approximate solution provided essential insights for nonlinear vibration analysis.

In the realm of various nonlinear system analysis methods, Nayfeh and Pai [11] compared several commonly used approaches. They highlighted that, in contrast to dealing with nonlinear partial differential equations and boundary conditions, the method of averaging (MOA) offers a simplification of the calculations by minimizing the reliance on algebra. Additionally, the method of multiple scales (MOMS) [12] proves to be a versatile approach applicable to vibration systems incorporating damping. In fluid pipelines, understanding the nonlinear vibrations induced by conveyed fluids is crucial. Czerwiński et al. [13] conducted an analysis on the influence of flow velocity and pipeline curvature on vibration modes and natural frequencies. Their findings revealed that tubes with larger radii and higher curvature exhibit higher natural frequencies. Additionally, fluid–structure coupling (FSC) significantly influences pipeline vibration. Wang and Wei [14] constructed a model for a nonlinear fluid transport beam with an elastic base to simulate the main body. Using Hamilton’s Principle and the method of multiple scales (MOMS), they calculated and analyzed fluid–solid coupling phenomena, exploring the potential occurrence of internal resonance. Syuhri et al. [15] emphasized that complex fluid–structure interactions often result in nonlinear dynamic behaviors in structures. Nonlinear modal analysis serves as a valuable tool for estimating, identifying, and quantifying these nonlinear behaviors. Malazi et al. [16] conducted simulations involving the deformation of a T-shaped flexible beam through model experiments, numerical simulations, and two-way fluid–structure coupling methods. Their observations indicated that the deformation and pressure in the flexible beam increased with speed, creating a pressure area at the top of the T-shaped beam, leading to heightened resistance at high speeds. Consequently, the T-shaped beam experienced greater deformation and higher stress levels. Wang and Chen [17] utilized nonlinear elastic beams to simulate fluid delivery pipe systems, delving into the impact of fluid–solid coupling and stretching effects. Employing Hamilton’s Principle, they derived the motion equation for fluid–solid coupling and employed MOMS to control the time scale, effectively analyzing the influence of parameter disturbances on fluid–structure coupling phenomena.

In recent years, many researchers have explored integrating vibration absorbers into vibration energy harvesting systems (VEHSs) to mitigate the vibration of the main structure and harness the electrical energy converted from vibrational energy. Pennisi et al. [18] studied a nonlinear energy sink (NES) with magnets, revealing that different vibration modes, such as cubic and bistable configurations, can be achieved based on the distance between additional magnets. The bistable configuration induces chaotic kinematic behavior, offering advantages for efficient energy absorption and harvesting. Wang et al. [19] introduced a novel dynamic model for a double-beam piezo-magneto-elastic nonlinear wind energy harvester (DBPME-WEH). Their research identified low, medium, and high wind speed ranges characterized by chaos and inter-well oscillations. Comparisons occurred regarding the effects of the length ratio and effective mass ratio of the beam. Magnetic interaction and the formation of a nonlinear bistable state were identified as key factors, with deeper energy wells leading to enhanced energy collection. Zang et al. [20] proposed a lever-type nonlinear energy sink (LNES) coupled with a levitation magneto-electric energy harvester (LMEH) as a nonlinear energy harvesting system. Additionally, Kecik [21] presented a magnetic levitation harvester (MLH) capable of simultaneously suppressing vibrations and collecting energy. The inclusion of a pendulum damper enabled the recovery of most of the energy in small amplitudes, leading to improved vibration damping when coupled with the device.

Chen et al. [22] introduced an asymmetric tristable nonlinear energy sink (ATNES) to enhance the vibration suppression efficiency. The ATNES, composed of oblique and vertical springs, achieves three stable states by adjusting the distance and stiffness asymmetry of the oblique springs. A comparative analysis of the three ATNES types demonstrates that the asymmetric design improves the vibration reduction efficiency. Rezaei et al. [23] proposed a bistable piezoelectric-based absorber (BPA) for simultaneous energy harvesting and vibration suppression. The BPA consists of a cantilever beam with PZT layers and two permanent magnets, generating bistability through magnetic interactions. The BPA is connected to a main vibrating beam. Numerical analyses in time and frequency domains reveal optimal performance during chaotic inter-well oscillations. The BPA demonstrates superior vibration suppression and energy harvesting over a wide frequency range compared to linear absorbers and nonlinear energy sinks. Guo et al. [24] compared the transient vibration suppression performance of nonlinear vibration absorbers (NVAs) with four magnet arrangements: two repulsive magnets (2RMNVA), two attractive magnets (2AMNVA), three repulsive magnets (3RMNVA), and three attractive magnets (3AMNVA). A dynamic model based on the dipole–dipole magnet interaction was established, and the key parameters were optimized for different excitation levels. Razaei et al. [25] investigated the dynamical responses and performances of mono-, bi-, and tristable nonlinear energy sinks (NESs) for simultaneous vibration suppression and energy harvesting. A multi-stable NES composed of a bimorph cantilever beam and magnet arrays was considered for a simply supported beam under harmonic excitation. Their results indicated that bi- and tristable absorbers perform well in strongly modulated responses. Energy-based analyses reveal that bistable absorbers outperform tristable ones in both vibration mitigation and energy harvesting. Rezaei et al. [26] investigated the tunable bistable magneto-piezoelastic absorber (BMPA), demonstrating its capability to achieve high-efficiency energy collection and vibration suppression. Their findings revealed that altering the magnet distance results in the absorber forming a periodic energy well, encompassing low-amplitude oscillations, chaotic large-scale vibrations between wells, and periodic high-amplitude oscillations. The bistable collector proved to be effective in significantly reducing vibrations and accumulating a substantial amount of energy. Yang et al. [27] highlighted the limitations of the traditional linear vibration energy harvesters, which are most effective near the resonance source, restricting the useful operating range for obtaining high output power. They proposed overcoming these limitations by opting for nonlinear energy harvesters. The nonlinear bistable system, characterized by two equilibrium points, features an energy function with two wells. Compared to nonlinear monostable energy harvesters, the bistable counterparts offer the advantage of large-amplitude output power from high-energy wells. Qian et al. [28] delved into the nonlinear dynamics of a simply supported beam with a nonlinear spring-inertial damper energy harvester to reduce the main resonance vibrations. Their study explored the influence of the shock absorber position, spring stiffness, and inertia on the beam’s dynamic effects, revealing that the maximum nonlinear frequency and highest nonlinear state occur when the nonlinear viscous absorber (NVA) is positioned in the middle of the beam. Liao et al. [29] examined a bistable piezoelectric energy harvester (BS-PEH) and developed kinetic equations and electromechanical coupling equations by integrating fluid-induced vibration. Lu et al. [30] investigated nonlinear energy harvesting from fluid-conveying piezoelectric pipes for self-powered sensing. Using electromechanical modeling and harmonic-balance analysis, the study explored the impact of fluid velocity on resonance peaks and output frequency response functions, demonstrating enhanced energy harvesting potential for self-powered sensing applications. Additionally, Rezaei et al. [31] introduced a tristable magneto-piezoelastic absorber (TMPA) that induces intra-well, non-periodic inter-well, and periodic inter-well oscillations. This design achieves both vibration reduction and energy collection effects. However, the research on the control and execution of tristability is still in its preliminary stage.

**Figure 1:**
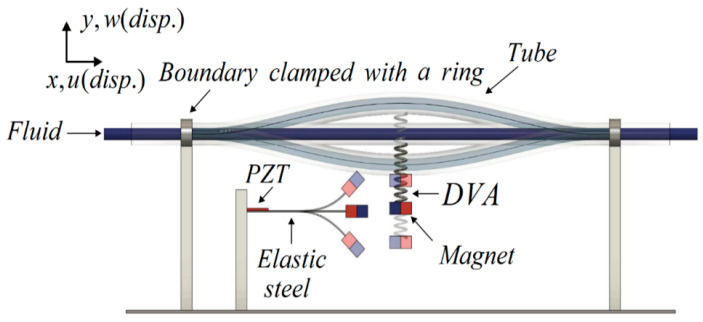
Diagram of the DVA and energy harvesting system for the flow tube.

Traditionally, the goal of vibration damping design has been to mitigate the vibrations in the main body for effective reduction. However, devices such as tuned mass dampers (TMDs) or dynamic vibration absorbers (DVAs) can inadvertently induce significant vibrations in the dampers themselves by absorbing the main body’s vibration. These secondary vibrations present an opportunity for repurposing by integrating an energy harvesting system. In this study, rather than solely adding a damper to achieve vibration reduction in a pipe system, a magnet is affixed to this dynamic vibration absorber device. The free end of its adjacent elastic steel sheet (elastic beam) is also equipped with magnets. This setup uses the mutual repulsion between magnets to excite the elastic steel to vibrate. Strategically placed at the root of the adjacent elastic steel sheet is a piezoelectric patch (PZT), further enhancing the setup’s functionality by generating electricity. The proposed system, referred to as the “flow tube vibration reduction and magneto-electric vibration energy harvesting system”, comprises three key components: a flow tube, a dynamic vibration absorber (DVA) with a magnet, and an elastic cantilever beam equipped with a piezoelectric patch (PZT). In this system, the mass in the vibration absorber is replaced with a magnet. The repulsive force of the magnet excites the adjacent fixed-free elastic steel fitted with a piezoelectric patch, creating a magnetic dipole–dipole interactions energy harvesting system (MDDI VEH) to enhance vibration energy collection (see Figure 1). The MDDI VEH system, although distinct from a traditional bistable VEH system, exhibits similarities in its operational principles. While the MDDI VEH system may generate less power compared to a bistable VEH system, it effectively enhances the amplitude of the elastic steel by continuously exciting the structure through magnetic repulsive forces. This excitation helps to maintain higher levels of oscillation, thereby facilitating energy harvesting and contributing to the longevity of the system. This study explores various magnet masses and spring constants of the DVA in the flow tube, aiming to determine the best position of the vibration absorber on the flow tube under different external load conditions to achieve the best damping effect on the fixed-free beam (flow tube) and maximize the electric energy generation.

**Figure 2:**
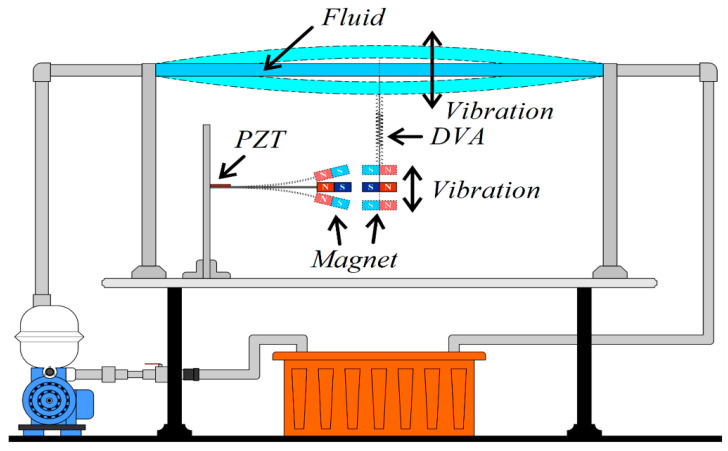
Conceptual experimental design of the fluid-structure coupled vibration damping and magneto-electric MDDI VEH system.

This research is structured into three parts: a theoretical model derivation, a theoretical and numerical analysis, and a simple experimental verification. (1) Theoretical Model Derivation: Utilizing Hamilton’s Principle, the equation for the nonlinear fluid–solid coupling elastic fixed-free beam with an additional DVA will be derived to simulate the motion of the fluid pipeline. The Biot–Savart Law will be employed to derive the repulsive force arising from the interaction between the magnets on the flow tube’s DVA and those on its neighboring elastic fixed-free beam. This damped pipe flow system will incorporate an elastic beam equipped with a piezoelectric patch (PZT), resulting in the creation of a comprehensive “flow tube vibration reduction and magnetic dipole–dipole interactions vibration energy harvesting system”. (2) Analytical and Numerical Analysis: The method of multiple scales (MOMS) will be employed to categorize the time domain into two scales: fast and slow. The separation of the variables will be applied to segregate the variables into the time and space domains. The boundary conditions will be used to determine the mode shape of the fixed-free beam. A fixed-points plot will be employed to analyze the frequency response and amplitude of the flow tube. The fourth-order Runge–Kutta method (RK-4) will be used to solve the equations governing the vibration of the flow tube and magneto-electric bistability, taking into account the mutual repulsion of magnets. This analysis will predict the vibration reduction effect of the vibration absorber and calculate the maximum efficiency of the electric energy conversion in the proposed system. (3) Verification of the Experiment: A simple experimental setup will be constructed, as depicted in the conceptual design schematic diagram in Figure 2. Fluid will be injected into the pipeline system using a water pump to simulate fluid flow, inducing vibration in the pipeline. The incorporation of a DVA aims to reduce the vibration in fluid pipelines. By leveraging the repulsive characteristics of magnets, the entire system will adopt a magnetic dipole–dipole interactions configuration, resulting in the vibration of the cantilever beam. The MDDI VEH system, created by this magnetic dipole–dipole interactions energy well configuration, will transfer the vibration energy from the DVA to the fixed-free cantilever beam, thereby enhancing the displacement and deformation. The installation of a piezoelectric patch at the root of the cantilever beam aims to maximize the deformation, thereby increasing the electric generation efficiency. 

## 2. Theoretical Model

This study utilizes a nonlinear Euler–Bernoulli beam to establish a theoretical model framework for fluid transportation pipelines. The pipeline is fixed at both ends (fixed-free), with a vibration absorber attached to the beam, where the mass of this vibration absorber is a magnet. Leveraging the mutual repulsion characteristics of magnets, the magnet at the end of another elastic beam is induced to vibrate, subsequently driving the elastic beam to undergo transverse vibrations. The elastic beam is supported akin to a cantilever beam, with the piezoelectric patch (PZT) positioned at the fixed end (root) of the elastic beam. The vibration induced by the fluid delivery pipe and the repulsive force of the magnet cause deformation in the elastic beam, leading the PZT at the root to generate electrical energy through deformation, serving the purpose of power generation. Hamilton’s Principle is employed to deduce the nonlinear motion of the fluid–structure coupling in the fluid delivery pipeline system. The flow velocity in the fluid pipeline and the equation of the vibration absorber coupled to this nonlinear system are considered. The Biot–Savart Law is then utilized to determine the Lorentz force exerted by the magnet on the vibration absorber on the adjacent elastic steel/VEH system. By coupling the piezoelectric equation of the PZT placed at the root of the elastic beam with the equation of the nonlinear cantilever beam, the Lorentz force of the magnet can disrupt the MDDI VEH system of the piezoelectric/elastic steel to enhance the power generation efficiency. A detailed analysis of the equations of motion will be conducted in the following sections to derive theoretical insights and evaluate the electric generation benefits of this nonlinear system.

### 2.1. Derivation of Nonlinear Fluid–Structure Coupling Equations of Motion

**Figure 3:**
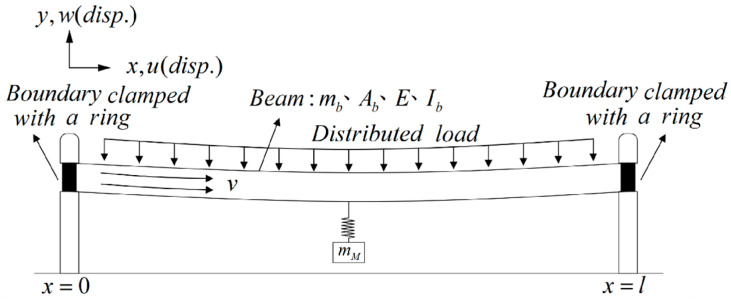
Schematic diagram of a flow pipeline system with a DVA.

Figure 3 illustrates a schematic diagram of a fluid delivery pipe system featuring a vibration absorber. In this conceptualization, we model a nonlinear fluid delivery pipe using the fixed-free beam as the theoretical framework. This versatile model can simulate various scenarios, including oil pipelines, factory ventilation and exhaust ducts, refrigeration and air conditioning ducts, submarine cables, submarine oil pipelines, and heat dissipation ducts in microelectromechanical systems. We assume the presence of a non-viscous fluid in the pipeline and consider the fluid within this nonlinear transport system to flow at a velocity of *v*(*t*), thereby generating substantial vibration energy within the pipeline. The main body is subject to a distributed force, with a vibration absorber coupled to the fluid delivery pipeline. In this context, *m_b_* represents the mass of the elastic beam per unit length, *A_b_* is the cross-sectional area of the elastic beam, *E* denotes the Young’s modulus of the elastic beam, *I_b_* is the moment of inertia of the elastic beam, and *m_M_* signifies the mass of the vibration absorber. 

**Figure 4:**
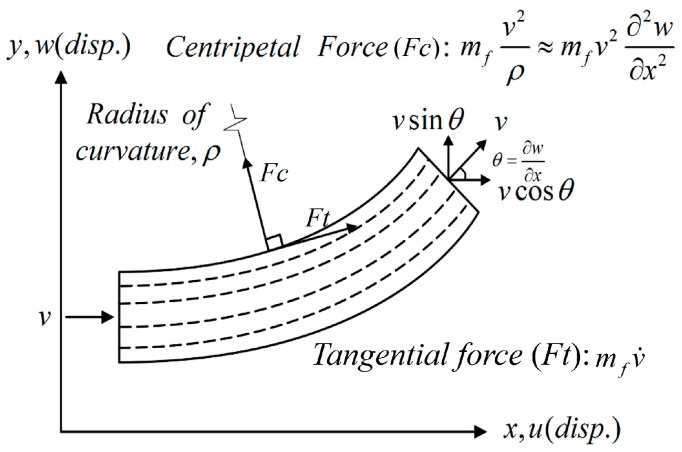
A small element in a fluid-structure coupling system.

We adopt the nonlinear Euler–Bernoulli beam as the model for the flow tube, assuming that this elastic beam retains its original size and shape even after deformation and remains in the same plane. Figure 4 illustrates a small element on the nonlinear elastic beam for analysis. In this context, the curvature of the beam is defined as $\kappa =\frac{1}{\rho }=\theta^{\prime }=w^{\prime \prime }$, ( )’ represents $\frac{\partial }{\partial x}$, and the centripetal force in the fluid flowing through the pipeline is defined as $m_{f}\frac{v^{2}}{\rho }\approx m_{f}v^{2}w^{\prime \prime }$, where *m_f_* represents the mass of the fluid. To establish the nonlinear strain of the beam, we employ the relationship between von Kármán’s nonlinear strain (η) and deformation (*u*, *w*) while retaining only the square term change of $w^{\prime }$. This results in obtaining the nonlinear strain of the beam as $\eta =u^{\prime }+\frac{1}{2}{\left(w^{\prime }\right)}^{2}+y(w^{\prime \prime })=\eta_{0}+\eta_{1}$, where $\eta_{0}=u^{\prime }+\frac{1}{2}{w^{\prime }}^{2}$ represents the tensile strain and $\eta_{1}=yw^{\prime \prime }$ represents the bending strain. In this representation, *y* denotes the coordinate system of the cross-section of the beam, with *u* and *w* representing the directions of the *x* and *y* axes, respectively. This study utilizes Hamilton’s Principle to derive the equation of motion for the fluid–solid coupling in this nonlinear system, expressing the kinetic energy (*T*) as

(1) $$T=\int_{0}^{l}(\frac{1}{2}m_{b}(\dot{u}+y{\dot{w}}^{\prime }{)}^{2}+\frac{1}{2}m_{b}{\dot{w}}^{2})dx+\int_{0}^{l}(\frac{1}{2}m_{f}{\left(\vec{V}\right)}^{2}+\frac{1}{2}m_{f}{\left(y{\dot{w}}^{\prime }\right)}^{2})dx+\frac{1}{2}\int_{0}^{l}m_{M}{\left({\dot{w}}_{M}\right)}^{2}dx$$

The first integral term represents the kinetic energy of the beam, the second integral term signifies the kinetic energy of the fluid, and the third integral term denotes the kinetic energy of the vibration absorber. ( )’ represents $\frac{\partial }{\partial x}$, and $(\;\dot{)}$ represents $\frac{\partial }{\partial t}$. Here, *m_b_* represents the mass of the beam, *m_f_* is the mass of the fluid, and *m_M_* is the mass of the vibration absorber. Additionally, *w_M_* represents the displacement of the vibration absorber, $w(l_{M},t)$ denotes the displacement on the beam position of *l_M_*, and *l_M_* represents the position where the vibration absorber is installed on the beam. The variables *u* and *w* symbolize the displacement of the elastic beam in the *x* and *y* directions, respectively. $y{\dot{w}}^{\prime }=y\dot{\theta }=y\omega$ is the velocity term, and $\vec{V}=(v\cos(\theta )+\dot{u})\vec{i}+(v\sin(\theta )+\dot{w})\vec{j}$ represents the relative velocity between the fluid and the beam, where the flow velocity *v* is a function of time. Then the potential energy of the system can be expressed as

(2) $$U=\int_{0}^{l}\frac{1}{2}EA_{b}\eta^{2}dx+\int_{0}^{l}\{-qw+m_{f}{\vec{V}}^{2}w^{\prime \prime }(w\cos(\theta )-u\sin(\theta ))+m_{f}\dot{v}(u\cos(\theta )+w\sin(\theta )\}dx+\int_{0}^{l}\frac{1}{2}k_{M}{\left(w_{M}-w(l_{M},t)\right)}^{2}dx$$

The first integral term corresponds to the nonlinear strain potential energy of the beam, the second integral term denotes the fluid potential energy, and the third integral term represents the potential energy of the vibration absorber. Here, *E* is the Young’s modulus of the elastic beam, and *A_b_* is the cross-sectional area of the beam. The variables *q*, $m_{f}{\vec{V}}^{2}w^{\prime \prime }$, $m_{f}\dot{v}$, θ, and *k_M_* represent a distributed simple harmonic external force, the centripetal force of the fluid acting on the beam, the tangential force of the fluid acting on the beam, the bending angle of the beam, and the spring constant of the vibration absorber, respectively. Subsequently, we utilize Taylor series expansion to expand cos(θ) and sin(θ), allowing the flow rate to be expressed as

(3) $$\vec{V}=(v(1-\frac{{w^{\prime }}^{2}}{2})+\dot{u})\vec{i}+(v(w^{\prime }-\frac{{w^{\prime }}^{3}}{6})+\dot{w})\vec{j})$$

Subsequently, apply Hamilton’s Principle and take the variation of both kinetic energy and potential energy. Introduce Equation (3) to yield the kinetic energy and potential energy variation integration as:

(4) $$\int_{0}^{T}\delta Tdt=-\int_{0}^{T}\int_{0}^{l}(m_{b}+m_{f})\ddot{u}\delta udxdt-\int_{0}^{T}\int_{0}^{l}((m_{b}+m_{f})\ddot{w}-(m_{b}+m_{f})y^{2}{\ddot{w}}^{\prime \prime })dxdt\delta w\;+\int_{0}^{T}\int_{0}^{l}m_{f}(v-\frac{1}{2}v{w^{\prime }}^{2}+\dot{u}+{\dot{u}}^{\prime }w^{\prime }w+\dot{u}w^{\prime \prime }w+\frac{2}{3}v^{\prime }{w^{\prime }}^{3}w-\frac{1}{6}v^{\prime }{w^{\prime }}^{5}w-\frac{1}{6}vw^{\prime \prime }{w^{\prime }}^{4}w\;-{\dot{w}}^{\prime }w+{\dot{w}}^{\prime }{w^{\prime }}^{2}w+\dot{w}w^{\prime \prime }w^{\prime }w+2vw^{\prime \prime }{w^{\prime }}^{2}w)\delta vdxdt\;+\int_{0}^{T}\int_{0}^{l}m_{f}(v{\dot{u}}^{\prime }w^{\prime }+v\dot{u}w^{\prime \prime }+\frac{2}{3}v^{\prime }v{w^{\prime }}^{3}2v^{2}w^{\prime \prime }{w^{\prime }}^{2}-\frac{1}{6}v^{\prime }v{w^{\prime }}^{5}-\frac{5}{6}v^{2}w^{\prime \prime }{w^{\prime }}^{4}-\dot{v}w^{\prime }+\frac{1}{6}\dot{v}w^{\prime }+v\dot{w}w^{\prime \prime }w^{\prime }\;+\frac{3}{2}v{\dot{w}}^{\prime }{w^{\prime }}^{2}-2v{\dot{w}}^{\prime })\delta wdxdt\;-\int_{0}^{T}\int_{0}^{l}m_{M}{\ddot{w}}_{M}\delta wdxdt+\int_{0}^{T}\int_{0}^{l}m_{M}{\ddot{w}}_{M}\delta w_{M}dxdt$$

(5) $$\int_{0}^{T}\delta Udt=\int_{0}^{T}\int_{0}^{l}EA_{b}(-u^{\prime \prime }-w^{\prime }w^{\prime \prime })\delta udxdt+\int_{0}^{T}\int_{0}^{l}-q\delta wdxdt\;+\int_{0}^{T}\int_{0}^{l}EA_{b}(-u^{\prime \prime }-w^{\prime }w^{\prime \prime }-\frac{3}{2}{w^{\prime }}^{2}w^{\prime \prime }+y^{2}w^{iv})\delta wdxdt\;+\int_{0}^{T}\int_{0}^{l}m_{f}(\dot{v}-v^{2}w^{\prime \prime }w^{\prime }+\frac{1}{6}v^{2}w^{\prime \prime }{w^{\prime }}^{3}-\frac{1}{2}\dot{v}{w^{\prime }}^{2})\delta udxdt\;+\int_{0}^{T}\int_{0}^{l}m_{f}(v^{2}w^{\prime \prime }+\frac{1}{2}v^{2}w^{\prime \prime }{w^{\prime }}^{2}+\dot{v}w^{\prime }-\frac{1}{6}\dot{v}{w^{\prime }}^{3})\delta wdxdt\;+\int_{0}^{T}\int_{0}^{l}k_{M}(-w+w_{M})\delta wdxdt+\int_{0}^{T}\int_{0}^{l}k_{M}(w-w_{M})\delta w_{M}dxdt$$

The coupled equation for the elastic beam involved in fluid transport in the *u*-direction can be derived as

(6) $$(m_{b}+m_{f})\ddot{\bar{u}}-EA_{b}({\bar{u}}^{\prime \prime }+{\bar{w}}^{\prime \prime }{\bar{w}}^{\prime })+m_{f}(\dot{\bar{v}}-{\bar{v}}^{2}{\bar{w}}^{\prime \prime }{\bar{w}}^{\prime }+\frac{1}{6}{\bar{v}}^{2}{\bar{w}}^{\prime \prime }{{\bar{w}}^{\prime }}^{3}-\frac{1}{2}\dot{\bar{v}}{{\bar{w}}^{\prime }}^{2})=0$$

The coupled motion equation of the elastic beam for fluid transport in the *w*-direction is obtained as

(7) $$(m_{b}+m_{f})\ddot{\bar{w}}-(m_{b}+m_{f})y^{2}{\ddot{\bar{w}}}^{\prime \prime }+EA_{b}y^{2}{\bar{w}}^{\prime \prime \prime \prime }-EA_{b}({\bar{u}}^{\prime \prime }{\bar{w}}^{\prime }+{\bar{u}}^{\prime }{\bar{w}}^{\prime \prime }+\frac{3}{2}{\bar{w}}^{\prime \prime }{{\bar{w}}^{\prime }}^{2})+m_{f}(-\bar{v}\dot{\bar{u}}{\bar{w}}^{\prime }-\bar{v}\dot{\bar{u}}{\bar{w}}^{\prime \prime }-\frac{2}{3}\bar{v}{\bar{v}}^{\prime }{{\bar{w}}^{\prime }}^{3}-\frac{5}{2}{\bar{v}}^{2}{\bar{w}}^{\prime \prime }{{\bar{w}}^{\prime }}^{2}\;+\frac{1}{6}\bar{v}{\bar{v}}^{\prime }{{\bar{w}}^{\prime }}^{5}+\frac{5}{6}{\bar{v}}^{2}{\bar{w}}^{\prime \prime }{{\bar{w}}^{\prime }}^{4}+2\dot{\bar{v}}{\bar{w}}^{\prime }-\frac{1}{3}\dot{\bar{v}}{{\bar{w}}^{\prime }}^{3}-\bar{v}\dot{\bar{w}}{\bar{w}}^{\prime }{\bar{w}}^{\prime \prime }-\frac{3}{2}\bar{v}{\dot{\bar{w}}}^{\prime }{{\bar{w}}^{\prime }}^{2}+2\bar{v}{\dot{\bar{w}}}^{\prime }+{\bar{v}}^{2}{\bar{w}}^{\prime \prime })-q=0$$

And the vibration absorber equation is shown as

(8) $$m_{M}{\ddot{\bar{w}}}_{M}-k_{M}(\bar{w}-{\bar{w}}_{M})=0$$

In Equation (7), *EA_b_y*^2^ = *EI_b_* represents the stiffness of the beam, while $m_{b}y^{2}=I_{b}$ and *m_f_y*^2^*=I_f_* denote the mass moments of inertia of the beam and the fluid, respectively. While general damping can be incorporated directly into the equation of motion using Newton’s Second Law, in the coupled fluid–structure equation, we assume that energy dissipation can be modeled by small, uncoupled, viscous dampers. The same method is also applied in the paper by Pai et al. [11,32]. By incorporating the damping terms $\mu_{u}\dot{u}$ and $\mu_{w}\dot{w}$ into Equations (6) and (7), we obtain the coupled equation

(9) $$(m_{b}+m_{f})\ddot{\bar{u}}-EA_{b}({\bar{u}}^{\prime \prime }+{\bar{w}}^{\prime \prime }{\bar{w}}^{\prime })+{\bar{\mu }}_{u}\dot{\bar{u}}+m_{f}(\dot{\bar{v}}-{\bar{v}}^{2}{\bar{w}}^{\prime \prime }{\bar{w}}^{\prime }+\frac{1}{6}{\bar{v}}^{2}{\bar{w}}^{\prime \prime }{{\bar{w}}^{\prime }}^{3}-\frac{1}{2}\dot{\bar{v}}{{\bar{w}}^{\prime }}^{2})=0$$

(10) $$(m_{b}+m_{f})\ddot{\bar{w}}-(I_{b}+I_{f}){\ddot{\bar{w}}}^{\prime \prime }+EI_{b}{\bar{w}}^{iv}-EA_{b}({\bar{u}}^{\prime \prime }{\bar{w}}^{\prime }+{\bar{u}}^{\prime }{\bar{w}}^{\prime \prime }+\frac{3}{2}{\bar{w}}^{\prime \prime }{{\bar{w}}^{\prime }}^{2})+{\bar{\mu }}_{w}\dot{\bar{w}}+m_{f}(-{\dot{\bar{u}}}^{\prime }{\bar{w}}^{\prime }\bar{v}-\dot{\bar{u}}{\bar{w}}^{\prime \prime }\bar{v}-\frac{2}{3}{\bar{v}}^{\prime }\bar{v}{{\bar{w}}^{\prime }}^{3}\;-\frac{5}{2}{\bar{v}}^{2}{\bar{w}}^{\prime \prime }{{\bar{w}}^{\prime }}^{2}+\frac{1}{6}\bar{v}{\bar{v}}^{\prime }{{\bar{w}}^{\prime }}^{5}+\frac{5}{6}{\bar{v}}^{2}{\bar{w}}^{\prime \prime }{{\bar{w}}^{\prime }}^{4}+2\dot{\bar{v}}{\bar{w}}^{\prime }-\frac{1}{3}\dot{\bar{v}}{{\bar{w}}^{\prime }}^{3}-\bar{v}\dot{\bar{w}}{\bar{w}}^{\prime }{\bar{w}}^{\prime \prime }-\frac{3}{2}\bar{v}{\dot{\bar{w}}}^{\prime }{{\bar{w}}^{\prime }}^{2}+2\bar{v}\dot{\bar{w}}+{\bar{v}}^{2}{\bar{w}}^{\prime \prime })-q=0$$

For ease of subsequent calculation and analysis, we proceed to nondimensionalize Equations (9) and (10) and Equation (8). The dimensionless definitions of each coefficient are provided in Appendix A. To simplify further analysis, this study employs the same symbols as those with dimensions to represent the results following nondimensional transformation. Thus, the equations of motion become

(11) $$\ddot{u}-(u^{\prime \prime }+w^{\prime \prime }w^{\prime })+\mu_{u}\dot{u}+M(\dot{v}-v^{2}w^{\prime \prime }w^{\prime }+\frac{1}{6}v^{2}w^{\prime \prime }{w^{\prime }}^{3}-\frac{1}{2}\dot{v}{w^{\prime }}^{2})=0$$

(12) $$\ddot{w}-I\ddot{w}+\mu_{w}\dot{w}+\omega^{2}w^{iv}-(u^{\prime \prime }w^{\prime }+u^{\prime }w^{\prime \prime }+\frac{3}{2}w^{\prime \prime }{w^{\prime }}^{2})+M(-\dot{u^{\prime }}w^{\prime }v-\dot{u}w^{\prime \prime }v-\frac{2}{3}v^{\prime }v{w^{\prime }}^{3}-\frac{5}{2}v^{2}w^{\prime \prime }{w^{\prime }}^{2}\;+\frac{1}{6}vv^{\prime }{w^{\prime }}^{5}+\frac{5}{6}v^{2}w^{\prime \prime }{w^{\prime }}^{4}+2\dot{v}w^{\prime }-\frac{1}{3}\dot{v}{w^{\prime }}^{3}-v\dot{w}w^{\prime }w^{\prime \prime }-\frac{3}{2}v\dot{w^{\prime }}{w^{\prime }}^{2}+2v\dot{w}+v^{2}w^{\prime \prime })-qe^{i\Omega \tau }=0$$

(13) $${\ddot{w}}_{M}-{\omega_{x}}^{2}(w-w_{M})=0$$

Among them, $\omega_{u}$, $\omega_{w}$ and $\omega_{M}$ represent the *u*, *w* directions and the dimensionless frequency of the vibration absorber respectively, and let $\omega_{u}=\sqrt{\frac{EA_{b}}{(1+M)l^{2}}}$, $\bar{M}=\frac{M}{1+M}$, $I=\frac{\left(I_{b}+I_{f}\right)}{m_{b}(1+M)l^{2}}$, $\omega_{w}=\sqrt{\frac{EI_{b}}{m_{b}(1+M)l^{4}}}$, $\Omega =\frac{\omega_{f}}{\omega_{u}}$, $\omega_{x}=\sqrt{\frac{{\bar{k}}_{m}}{m_{D}}}$, $m_{D}=\frac{m_{M}}{m_{b}}$, ${\bar{k}}_{m}=\frac{k_{m}}{m_{b}}$, The mass of the elastic beam is $m_{b}$, the mass of the vibration absorber is $m_{M}$, and *m_f_* is the fluid mass, and *M* $=\frac{m_{f}}{m_{b}}$. Since both ends of the elastic beam are fixed-free, the dimensionless boundary conditions can be formulated as

(14) u(0,τ)=u(1,τ)=0

(15) $$w(0,\tau )=w^{\prime }(0,\tau )=w(1,\tau )=w^{\prime }(1,\tau )$$

τ is dimensionless time. Next, we assume that there is a uniform flow field in the fluid delivery pipe, and the deformation of the elastic beam in the *u* direction is a steady state, and then Equation (11) can be converted into

(16) $$u^{\prime \prime }=-w^{\prime \prime }w^{\prime }+M(\dot{v}-v^{2}w^{\prime \prime }w^{\prime }+\frac{1}{6}v^{2}w^{\prime \prime }{w^{\prime }}^{3}-\frac{1}{2}\dot{v}{w^{\prime }}^{2})$$

After integrating Equation (16), we can obtain $u^{\prime }$ and *u*:

(17) $$u^{\prime }=-(\frac{1}{2}+\frac{1}{2}Mv^{2}){w^{\prime }}^{2}+M\dot{v}x+\frac{1}{24}Mv^{2}{w^{\prime }}^{4}-\frac{1}{2}M\dot{v}\int_{0}^{x}{w^{\prime }}^{2}dx+C_{1}(x)$$

(18) $$u=-(\frac{1}{2}+\frac{1}{2}Mv^{2})\int_{0}^{x}{w^{\prime }}^{2}dx+\frac{1}{2}M\dot{v}x^{2}+\frac{1}{24}Mv^{2}\int_{0}^{x}{w^{\prime }}^{4}dx-\frac{1}{2}M\dot{v}\int_{0}^{x}\int_{0}^{x}{w^{\prime }}^{2}dxdx+C_{1}(x)x+C_{2}(x)$$

Substituting the boundary conditions of *u* direction direction (Equation (14)) into Equation (18), we can obtain

(19) $$C_{2}(x)=0$$

(20) $$C_{1}(x)=(\frac{1}{2}+\frac{1}{2}Mv^{2})\int_{0}^{1}{w^{\prime }}^{2}dx+\frac{1}{2}M\dot{v}x+\frac{1}{24}Mv^{2}\int_{0}^{1}{w^{\prime }}^{4}dx-\frac{1}{2}M\dot{v}\int_{0}^{1}\int_{0}^{x}{w^{\prime }}^{2}dxdx$$

By substituting Equations (16)–(20) into Equation (12) and integrating them with Equation (13), we can derive the fluid–structure coupling motion equation in the *w* direction:

(21) $$\ddot{w}-I{\ddot{w}}^{\prime \prime }+\mu_{w}\dot{w}+\omega^{2}w^{iv}+\frac{1}{2}M\dot{v}w^{\prime \prime }\int_{0}^{1}{w^{\prime }}^{2}dx-(\frac{1}{2}+\frac{1}{2}Mv^{2})w^{\prime \prime }\int_{0}^{1}{w^{\prime }}^{2}dx+\frac{1}{2}M\dot{v}w^{\prime \prime }-M\dot{v}w^{\prime \prime }x\;+\frac{1}{24}Mv^{2}w^{\prime \prime }\int_{0}^{1}{w^{\prime }}^{4}dx-\frac{1}{2}M\dot{v}w^{\prime \prime }\int_{0}^{1}\int_{0}^{x}{w^{\prime }}^{2}dxdx+M(-v^{2}w^{\prime \prime }{w^{\prime }}^{2}+\frac{5}{8}v^{2}w^{\prime \prime }{w^{\prime }}^{4}+\dot{v}w^{\prime }\;-\frac{1}{6}\dot{v}{w^{\prime }}^{3}-v^{2}w^{\prime }w^{\prime \prime }\dot{w}-\frac{3}{2}v{w^{\prime }}^{2}{\dot{w}}^{\prime }+2v{\dot{w}}^{\prime }+v^{2}w^{\prime \prime })-qe^{i\Omega \tau }-{\omega_{x}}^{2}(w-w_{M})=0$$

### 2.2. Simulation and Analysis of Magnet Repulsion Force

There are several references that address the modeling of magnetic interactions using the dipole–dipole method. Fang et al. [33] employed the geometrical dipole–dipole method to model magnetic interactions within the tuned bistable nonlinear energy sink (TBNES). Fan et al. [34] introduced an oscillating substructure that magnetically interacts with a parametrically excited cantilever-based piezoelectric energy harvester, promoting easier snap-through transitions, broadening the operational frequency range, and enhancing energy harvesting efficiency. Mei [35] developed a magnetic-linkage nonlinear piezoelectric energy harvester (PEH) employing one vertical and two horizontal piezoelectric beams coupled by magnetic forces to create time-varying potential wells. Fan et al. [36] uniquely used a directly excited element as an external oscillation source to trigger large-amplitude oscillations in a parametrically excited element through magnetic coupling effects.

These studies primarily focus on excitation phenomena caused by magnetic repulsion to achieve greater energy conversion efficiency. Although detailed magnetic force simulations provide accurate magnetic repulsion, they often rely on extensive computational methods, which are not particularly beneficial for the simplified coupling equations needed for the pipeline and elastic beam in this paper. This paper aims to verify that the dynamic vibration absorber (DVA) can simultaneously achieve vibration reduction and energy conversion efficiency. An analytical function that is conducive to coupling with other structural equations is the first choice. We found that Wang et al. [37] and Leng et al. [38] used the Biot–Savart Law to determine the repulsion between magnets, and this method was also successfully applied in Wang et al. [17]. Therefore, this paper will use the Biot–Savart Law and the methods in Refs. [37,38] to determine the expression for the potential energy caused by the repulsion between magnets.

**Figure 5:**
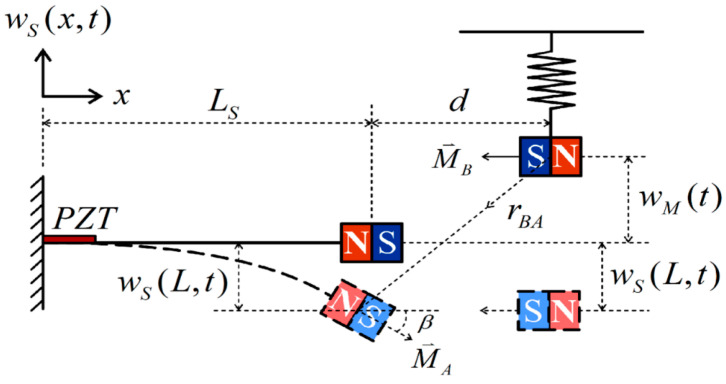
Schematic diagram of Lorentz force acting on a cantilever beam.

In Figure 5, ${\vec{r}}_{BA}$ represents the position vector between magnet *A* and magnet *B*, expressed as $-d\vec{i}-(w_{M}(t)+w_{S}(L_{M},t))\vec{j}$, where *d* is the distance between the two magnets. Additionally, $w_{M}$ denotes the displacement of the vibration absorber, $w_{S}(L_{M},t)=\phi (L_{M})\xi (t)$, and $\phi (L_{M})$ signifies the mode shape at $x=L_{M}$ position on the fixed-free beam (the flow tube). ξ(t) represents the generalized coordinates of the fixed-free beam (the flow tube), and ${\vec{M}}_{A}$ and ${\bar{M}}_{B}$ represent the magnetic moments of magnet *A* and magnet *B*, respectively. Furthermore, ${\vec{M}}_{A}={\bar{M}}_{A}{\bar{V}}_{A}\cos\beta \vec{i}+{\bar{M}}_{A}{\bar{V}}_{A}\sin\beta \vec{j}$, ${\vec{M}}_{B}=-{\bar{M}}_{B}{\bar{V}}_{B}\vec{i}$; ${\bar{M}}_{A}$ and ${\bar{M}}_{B}$ denote the magnitude of ${\vec{M}}_{A}$ and ${\vec{M}}_{B}$, respectively. ${\bar{V}}_{A}$ and ${\bar{V}}_{B}$ represent the volume of magnet *A* and *B*, respectively. The expression of the potential energy caused by the magnets, determined through the Biot–Savart Law, is as follows:

(22) $$U_{M}=\frac{\mu_{M}}{4\pi }{\vec{M}}_{A}(\frac{{\vec{M}}_{B}}{{\left\|{\vec{r}}_{BA}\right\|}_{2}^{3}}-\frac{3({\vec{M}}_{B}{\vec{r}}_{BA}){\vec{r}}_{BA}}{{\left\|{\vec{r}}_{BA}\right\|}_{2}^{5}})$$

In Equation (22), μM=4π×10−7Hm is the magnet constant, ${\left\|\;\right\|}_{2}$ is L2-normalization, ${\left\|x\right\|}_{2}=\sqrt{\left(\Sigma {x_{i}}^{2}\right)}$$=\sqrt{{x_{1}}^{2}+{x_{2}}^{2}+\cdots +{x_{i}}^{2}},$ $x={\vec{r}}_{BA}$. After organizing and calculating Equation (22), we can obtain

(23) $${\bar{U}}_{M}=\frac{\mu_{M}}{4\pi }{\vec{M}}_{A}(\frac{-{\bar{M}}_{B}{\bar{V}}_{B}\vec{i}{\left\|{\vec{r}}_{BA}\right\|}_{2}^{2}}{{\left\|{\vec{r}}_{BA}\right\|}_{2}^{5}}-\frac{3(-{\bar{M}}_{B}{\bar{V}}_{B}\vec{i}(-d\vec{i}-(w_{M}+w_{S})\vec{j}))(-d\vec{i}-(w_{M}+w_{S})\vec{j})}{{\left\|{\vec{r}}_{BA}\right\|}_{2}^{5}})\;=\frac{\mu_{M}{\bar{M}}_{A}{\bar{V}}_{A}{\bar{M}}_{B}{\bar{V}}_{B}[2d^{2}-{\left(w_{M}+w_{S}\right)}^{2}-3d(w_{M}+w_{S})]}{4\pi {\left[d^{2}+{\left(w_{M}+w_{S}\right)}^{2}\right]}^{5/2}}$$

Next, compute the Lorentz force applied by the magnet on the nonlinear cantilever beam. Divide the deformation on the cantilever beam into the spatial term and time term. Then, differentiate the time term. By setting $w_{S}=\phi_{S}(x)\xi_{S}(\tau )$, the following relationship can be established:

(24) $${\bar{F}}_{M}(t)=\frac{dU_{M}}{d\xi (t)}=\frac{\mu_{M}{\bar{M}}_{A}{\bar{V}}_{A}{\bar{M}}_{B}{\bar{V}}_{B}}{4\pi }\left\{\frac{5\phi_{S}(w_{M}+\phi_{S}\xi_{S})[{\left(w_{M}+\phi_{S}\xi_{S}\right)}^{2}+3\bar{d}(w_{M}+\phi_{S}\xi_{S})-2{\bar{d}}^{2}]}{{\left[{\left(w_{M}+\phi_{S}\xi_{S}\right)}^{2}+{\bar{d}}^{2}\right]}^{7/2}}\right.\;\left.-\frac{3\bar{d}\phi_{S}+2\phi_{S}(w_{M}+\phi_{S}\xi_{S})}{{\left[{\left(w_{M}+\phi_{S}\xi_{S}\right)}^{2}+{\bar{d}}^{2}\right]}^{5/2}}\right\}$$

Divide Equation (24) by $\frac{\bar{m}EI}{\rho A{\bar{l}}^{3}}$ to make it dimensionless, and use the same symbols to represent the dimensionless Lorentz force (*F_M_*); we can obtain

(25) $$F_{M}(t)=\frac{\mu_{M}M_{A}V_{A}M_{B}V_{B}}{4\pi }\left\{\frac{5\phi_{S}(w_{M}+\phi_{S}\xi_{S})[{\left(w_{M}+\phi_{S}\xi_{S}\right)}^{2}+3d(w_{M}+\phi_{S}\xi_{S})-2d^{2}]}{{\left[{\left(w_{M}+\phi_{S}\xi_{S}\right)}^{2}+d^{2}\right]}^{7/2}}\right.\;\left.-\frac{3d\phi_{S}+2\phi_{S}(w_{M}+\phi_{S}\xi_{S})}{{\left[{\left(w_{M}+\phi_{S}\xi_{S}\right)}^{2}+d^{2}\right]}^{5/2}}\right\}$$

Then, combining Equation (25) with Equation (13), the equation of the vibration absorber affected by Lorentz force can be obtained as follows:

(26) $${\ddot{w}}_{M}-{\omega_{x}}^{2}(w-w_{M})=F_{M}$$

### 2.3. Derivation of the Nonlinear Cantilever Beam Equation

This section presents a nonlinear equation for a fixed-free elastic steel beam, with the theoretical framework based on the nonlinear Euler–Bernoulli beam model for a cantilever beam. By incorporating boundary conditions and dimensionless time and space terms, we derive the equation of motion for this cantilever beam using principles such as Newton’s Second Law, Taylor’s expansion, and three-dimensional Euler angular coordinate conversion. For a more detailed derivation, please refer to Nayfeh and Pai [11] and Wang and Chu [39], from which the equation of motion for the beam can be obtained.

(27) $$m\ddot{\bar{u}}-EA{\bar{u}}^{\prime \prime }=EA(\frac{1}{2}{{\bar{w}}^{\prime }}^{2}-{\bar{u}}^{\prime }{{\bar{w}}^{\prime }}^{2}{)}^{\prime }+EI({\bar{w}}^{\prime }({\bar{w}}^{\prime \prime \prime }-{\bar{u}}^{\prime \prime \prime }{\bar{w}}^{\prime }-2{\bar{u}}^{\prime \prime }{\bar{w}}^{\prime \prime }-3{\bar{u}}^{\prime }{\bar{w}}^{\prime \prime \prime }){)}^{\prime }\;m\ddot{\bar{w}}+EI{\bar{w}}^{iv}-EA({\bar{u}}^{\prime }{\bar{w}}^{\prime }-{{\bar{u}}^{\prime }}^{2}{\bar{w}}^{\prime }+\frac{1}{2}{{\bar{w}}^{\prime }}^{3}{)}^{\prime }+{\bar{F}}_{M}-EI({\bar{u}}^{\prime }{\bar{w}}^{\prime \prime \prime }+({\bar{u}}^{\prime }{\bar{w}}^{\prime }{)}^{\prime \prime })$$

(28) $$-({{\bar{u}}^{\prime }}^{2}-{{\bar{w}}^{\prime }}^{2}){\bar{w}}^{\prime \prime \prime }-{\bar{u}}^{\prime }({\bar{u}}^{\prime }{\bar{w}}^{\prime }{)}^{\prime \prime }-({{\bar{u}}^{\prime }}^{2}{\bar{w}}^{\prime }-\frac{1}{3}{{\bar{w}}^{\prime }}^{3}{)}^{\prime \prime }{)}^{\prime }=0$$

Here, *m* represents the mass of the cantilever beam, *E* is Young’s modulus, *A* is the cross-sectional area of the beam, *I* is the moment of inertia of the beam, ${\bar{F}}_{M}$ denotes the Lorentz force influenced by the magnet, and $\bar{u}$ and $\bar{w}$ represent deformations in the *x* and *y* directions, respectively. PZT is assumed to be perfectly bonded to the elastic beam, and the PZT, elastic steel, and magnet are ideally integrated. Subsequently, the boundary conditions of this cantilever beam are applied. Given that $I_{m}$ is the mass moment of inertia, its boundary conditions can be specified as $\bar{w}(0,\bar{\tau })=0$, ${\bar{w}}^{\prime }(0,\bar{\tau })=0$, $EI{\bar{w}}^{\prime \prime }(\bar{l},\bar{\tau })=I_{m}\alpha^{4}a^{4}{\bar{w}}^{\prime }(\bar{l},\bar{\tau })$, $EI{\bar{w}}^{\prime \prime \prime }(\bar{l},\bar{\tau })=$ $-\bar{M}\alpha^{4}a^{4}\bar{w}(\bar{l},\bar{\tau })$. Using the boundary conditions of *u* (Equation (14), considering the steady state condition, integrating Equation (27), and substituting into Equation(28), we can obtain

(29) $$m\ddot{\bar{w}}+{\bar{\mu }}_{b}\dot{\bar{w}}+EI({\bar{w}}^{\prime \prime \prime }+{\bar{w}}^{\prime }{{\bar{w}}^{\prime \prime }}^{2}+{\bar{w}}^{\prime \prime \prime }{{\bar{w}}^{\prime }}^{2}{)}^{\prime }=-\frac{1}{2}m({\bar{w}}^{\prime }\int_{\bar{l}}^{\bar{x}}(\int_{0}^{\bar{x}}{{\bar{w}}^{\prime }}^{2}d\bar{x})d\bar{x}{)}^{\prime }+{\bar{F}}_{M}$$

Then the dimensionless equation of this nonlinear cantilever beam can be obtained:

(30) $$\ddot{w}+\mu_{b}\dot{w}+w^{iv}+{w^{\prime \prime }}^{3}+w^{iv}{w^{\prime }}^{2}+4w^{\prime }w^{\prime \prime \prime }w^{\prime \prime }=-\frac{1}{2}(w^{\prime }\int_{1}^{x}(\int_{0}^{x}{\ddot{w^{\prime }}}^{2}dx)dx{)}^{\prime }+F_{M}$$

where $\mu_{b}$ is the dimensionless damping coefficient of this beam.

It is noted that the elastic beam equipped with a PZT considered in this paper is regarded as a passive device. This means it is assumed that the magnet at the free end is excited solely by the magnetic force of the magnet installed on the DVA, which drives the elastic beam to vibrate and allows the PZT to generate voltage. No other external force is exerted on this elastic beam. This study does not consider the magnetic repulsive force interaction between the elastic beam’s magnet and the DVA’s magnet, nor does it account for any secondary excitation of the flow tube due to this magnetic repulsive force interaction.

### 2.4. Establishment of Theoretical Model of Piezoelectric Equation

In this section, we will formulate the piezoelectric equation for the piezoelectric patch (PZT), focusing on the Coulomb force exerted by the PZT on the nonlinear cantilever beam. Referring to Rajora [40], we can ascertain that the piezoelectric equation for the piezoelectric sheet under pressure is as follows:

(31) $$C_{p}\dot{V}+\frac{1}{{\bar{R}}_{p}}V+\int_{\bar{a}}^{\bar{b}}eh_{p}t_{h}{\dot{\bar{w}}}^{\prime \prime }d\bar{x}=0$$

where *V* is the voltage, *C_p_* is the capacitance of the piezoelectric patch, ${\bar{R}}_{p}$ denotes the load resistance, *e* stands for the dielectric constant, *h_p_* represents the length of the PZT, and *t_h_* is the thickness of the PZT. The variables $\bar{a}$ and $\bar{b}$ respectively denote the positions of the two ends of the PZT. Given that we position the PZT at the root of the cantilever beam, $\bar{a}$ = 0 and $\bar{b}$ = the length of the PZT. Subsequently, the piezoelectric equation is expressed as

(32) $$\int_{\bar{a}}^{\bar{b}}eh_{p}t_{h}{\dot{\bar{w}}}^{\prime \prime }d\bar{x}\frac{V}{h_{p}}=(et_{h}\int_{\bar{a}}^{\bar{b}}\dot{{\bar{w}}^{\prime \prime }}d\bar{x})V=C_{f}(\int_{\bar{a}}^{\bar{b}}\dot{{\bar{w}}^{\prime \prime }}d\bar{x})V$$

Here, *C_f_* represents the piezoelectric coupling coefficient. We have transformed Equation (31) into a dimensionless form. After performing calculations and arranging terms, and employing the same variables as in the dimensional equation, the resulting dimensionless equation is expressed as

(33) $$\dot{v}+R_{p}v+\hat{k}\int_{a}^{b}(\dot{w^{\prime \prime }})dx=0$$

where *v = V/C_f_*, $R_{p}=1/{\bar{R}}_{p}C_{p}\omega$, $\hat{k}=eh_{p}t_{h}/C_{p}C_{f}$, $a=\bar{a}/l$, $b=\bar{b}/l$, $(\;\dot{)}=d/d\tau$$=d/dT_{0}$. The dimensionless voltage can be obtained as

(34) $$v=-\frac{\hat{k}}{e^{R_{p}\tau }}\int_{0}^{\tau }(\int_{a}^{b}{\dot{w}}^{\prime \prime }dx)e^{R_{p}\tau }d\tau$$

Dividing Equation (34) by $lm\omega^{2}$, letting $\eta^{2}={C_{f}}^{2}/lm\omega^{2}$, and after sorting out, we can obtain the dimensionless external force (Coulomb force) function:

(35) $$\frac{C_{f}^{2}(\int_{a}^{b}w^{\prime \prime }dx)v}{lm\omega^{2}}=\eta^{2}(\int_{a}^{b}w^{\prime \prime }dx)v\;=\eta^{2}(\int_{a}^{b}w^{\prime \prime }dx)(-\frac{\hat{k}}{e^{R_{p}\tau }}\int_{0}^{\tau }(\int_{a}^{b}{\dot{w}}^{\prime \prime }dx)e^{R_{p}\tau }d\tau )=-\frac{\hat{k}\eta^{2}}{e^{R_{p}\tau }}\int_{a}^{b}w^{\prime \prime }dx\int_{0}^{\tau }(\int_{a}^{b}{\dot{w}}^{\prime \prime }dx)e^{R_{p}\tau }d\tau$$

Adding the external force term of the PZT to Equation (30), we can obtain

(36) $$\ddot{w}+\mu_{b}\dot{w}+w^{iv}+{w^{\prime \prime }}^{3}+w^{iv}{w^{\prime }}^{2}+4w^{\prime }w^{\prime \prime \prime }w^{\prime \prime }-\frac{\hat{k}\eta^{2}}{e^{R_{p}\tau }}\int_{a}^{b}w^{\prime \prime }dx\int_{0}^{\tau }(\int_{a}^{b}{\dot{w}}^{\prime \prime }dx)e^{R_{p}\tau }d\tau \;=-\frac{1}{2}(w^{\prime }\int_{1}^{x}(\int_{0}^{x}{\ddot{w^{\prime }}}^{2}dx)dx{)}^{\prime }+F_{M}$$

Among them, *F_M_* (please refer to Equation (25)) is the Lorentz force exerted on the nonlinear piezoelectric cantilever beam by the vibration absorber calculated using the Biot–Savart Law.

## 3. Theoretical Analysis

### 3.1. Method of Multiple Scales (MOMS)

The MOMS method divides time into two scales—fast and slow. Let $T_{0}=\tau$, $T_{1}=\varepsilon^{2}\tau$, where $T_{0}$ is the term of fast time scale and *T*_1_ denotes the slow scale of time. The *w* can be expressed as

(37) $$w(x,\tau ,\varepsilon )=\varepsilon w_{0}(x,T_{0},T_{1},T_{2}\cdots )+\varepsilon^{3}w_{1}(x,T_{0},T_{1},T_{2}\cdots )$$

Among them, ε is the perturbation term, which is regarded as a very small value, neglecting the higher-order terms above $\varepsilon^{4}$, and substituting Equation (37) into Equation (21), the nonlinear equation can be expressed as a multi-time-scale equation:

(38) $$\left(\varepsilon \frac{\partial^{2}w_{0}}{\partial {T_{0}}^{2}}+\varepsilon^{3}\frac{\partial^{2}w_{1}}{\partial {T_{0}}^{2}}+2\varepsilon^{3}\frac{\partial^{2}w_{0}}{\partial T_{0}\partial T_{1}})-I(\varepsilon \frac{\partial^{2}w_{0}}{\partial {T_{0}}^{2}}+\varepsilon^{3}\frac{\partial^{2}w_{1}}{\partial {T_{0}}^{2}}+2\varepsilon^{3}\frac{\partial^{2}w_{0}}{\partial T_{0}\partial T_{1}}\right)\;+\mu_{w}(\;\varepsilon \frac{\partial w_{0}}{\partial T_{0}}+\varepsilon^{3}\frac{\partial w_{1}}{\partial T_{0}}+\varepsilon^{3}\frac{\partial w_{0}}{\partial T_{1}})+\omega^{2}(\varepsilon {w_{0}}^{iv}+\varepsilon^{3}{w_{1}}^{iv})+\frac{1}{2}M\dot{v}(\varepsilon {w_{0}}^{\prime \prime }+\varepsilon^{3}{w_{1}}^{\prime \prime }){\int_{0}^{1}\left(\varepsilon {w_{0}}^{\prime }\right)}^{2}dx\;-(\frac{1}{2}+\frac{1}{2}Mv^{2})(\varepsilon {w_{0}}^{\prime \prime }+\varepsilon^{3}{w_{1}}^{\prime \prime }){\int_{0}^{1}\left(\varepsilon {w_{0}}^{\prime }\right)}^{2}dx+\frac{1}{2}M\dot{v}(\varepsilon {w_{0}}^{\prime \prime }+\varepsilon^{3}{w_{1}}^{\prime \prime })-M\dot{v}(\varepsilon {w_{0}}^{\prime \prime }+\varepsilon^{3}{w_{1}}^{\prime \prime })x\;+\frac{1}{24}Mv^{2}(\varepsilon {w_{0}}^{\prime \prime }+\varepsilon^{3}{w_{1}}^{\prime \prime })\int_{0}^{1}{\left(\varepsilon {w_{0}}^{\prime }\right)}^{4}-\frac{1}{2}M\dot{v}(\varepsilon {w_{0}}^{\prime \prime }+\varepsilon^{3}{w_{1}}^{\prime \prime })\int_{0}^{1}\int_{0}^{\bar{x}}{\left(\varepsilon {w_{0}}^{\prime }\right)}^{2}dxd\bar{x}\;-Mv^{2}(\varepsilon {w_{0}}^{\prime \prime }+\varepsilon^{3}{w_{1}}^{\prime \prime }){\left(\varepsilon {w_{0}}^{\prime }\right)}^{2}+\frac{5}{8}Mv^{2}(\varepsilon {w_{0}}^{\prime \prime }+\varepsilon^{3}{w_{1}}^{\prime \prime }){\left(\varepsilon {w_{0}}^{\prime }\right)}^{4}+M\dot{v}(\varepsilon {w_{0}}^{\prime }+\varepsilon^{3}{w_{1}}^{\prime })\;-\frac{1}{6}M\dot{v}{\left(\varepsilon {w_{0}}^{\prime }\right)}^{3}-Mv^{2}(\varepsilon {w_{0}}^{\prime }+\varepsilon^{3}{w_{1}}^{\prime })(\varepsilon {w_{0}}^{\prime \prime }+\varepsilon^{3}{w_{1}}^{\prime \prime })(\varepsilon \frac{\partial w_{0}}{\partial T_{0}}+\varepsilon^{3}\frac{\partial w_{1}}{\partial T_{0}}+\varepsilon^{3}\frac{\partial w_{0}}{\partial T_{1}})\;-\frac{3}{2}Mv{\left(\varepsilon {w_{0}}^{\prime }\right)}^{2}(\varepsilon \frac{\partial {w_{0}}^{\prime }}{\partial T_{0}}+\varepsilon^{3}\frac{\partial {w_{1}}^{\prime }}{\partial T_{0}}+\varepsilon^{3}\frac{\partial {w_{0}}^{\prime }}{\partial T_{1}})+2Mv(\varepsilon \frac{\partial {w_{0}}^{\prime }}{\partial T_{0}}+\varepsilon^{3}\frac{\partial {w_{1}}^{\prime }}{\partial T_{0}}+\varepsilon^{3}\frac{\partial {w_{0}}^{\prime }}{\partial T_{1}})\;+Mv^{2}(\varepsilon {w_{0}}^{\prime \prime }+\varepsilon^{3}{w_{1}}^{\prime \prime })-qe^{i\Omega \tau }-\omega_{x}(\varepsilon w_{0}+\varepsilon^{3}w_{1}-\varepsilon w_{M})\delta (x-l_{M})=0$$

Let the order of $qe^{i\Omega \tau }$ be $\varepsilon^{3}$, where $\Omega =\omega_{m}+\varepsilon \sigma$ denotes the frequency of external load, the order of $v^{2}$, $\mu_{w}$ and $\omega_{x}$ is $\varepsilon^{2}$, and the order of v and time derivite of v is $\varepsilon^{1}$, and list the time scale equations of $\varepsilon^{1}$ and $\varepsilon^{3}$, respectively, to facilitate subsequent analysis, and then the equation composed of the order of $\varepsilon^{1}$ is

(39) $$\frac{\partial^{2}w_{0}}{\partial {T_{0}}^{2}}-I\frac{\partial^{2}{w_{0}}^{\prime \prime }}{\partial {T_{0}}^{2}}+\omega^{2}{w_{0}}^{iv}=0$$

the equation composed of the order of $\varepsilon^{3}$ is

(40) $$\frac{\partial^{2}w_{1}}{\partial {T_{0}}^{2}}-I\frac{\partial^{2}{w_{1}}^{\prime \prime }}{\partial {T_{0}}^{2}}+\omega^{2}{w_{1}}^{iv}=-2\frac{\partial^{2}w_{0}}{\partial T_{0}\partial T_{1}}+2I\frac{\partial^{2}{w_{0}}^{\prime \prime }}{\partial T_{0}\partial T_{1}}-\mu_{w}\frac{\partial w_{0}}{\partial T_{0}}+\frac{1}{2}{w_{0}}^{\prime \prime }\int_{0}^{1}{\left({w_{0}}^{\prime }\right)}^{2}dx-Mv^{2}{w_{0}}^{\prime \prime }+qe^{i\Omega \tau }+{\omega_{x}}^{2}(w_{0}-w_{M})\delta (x-l_{M})$$

### 3.2. Analysis of Fluid–Solid Coupling Elastic Beam

In this section, we use the method of separation of variables to divide the deformation in the system into spatial and time domains, where X(x) is the space term and Y(τ) is the time term. We define the displacement *w*_0_ as

(41) $$w_{0}=X(x)Y(\tau )$$

And let γ be the eigenvalue of the beam, and then the general solution of *X* is

(42) $$X(x)=E_{1}\sin(\gamma x)+E_{2}\cos(\gamma x)+E_{3}\sinh(\gamma x)+E_{4}\cosh(\gamma x)$$

By using the boundary conditions (Equation (15), the characteristic equation of the elastic beam can be obtained as

(43) cos(γl)cosh(γl)=1

The mode shape is expressed as

(44) $$\phi_{n}(x)=-\frac{\cos(\gamma_{n}l)-\cosh(\gamma_{n}l)}{\sin(\gamma_{n}l)-\sinh(\gamma_{n}l)}\sin(\gamma_{n}x)+\cos(\gamma_{n}x)+\frac{\cos(\gamma_{n}l)-\cosh(\gamma_{n}l)}{\sin(\gamma_{n}l)-\sinh(\gamma_{n}l)}\sinh(\gamma_{n}x)-\cosh(\gamma_{n}x)$$

The subscript *n* = 1, 2, 3…

### 3.3. System Frequency Analysis

In this section, fixed-points plots are utilized to analyze the frequency response of each mode. Subsequently, time responses are employed to verify the accuracy of the fixed-points plots. First, the equation composed of the order of $\varepsilon^{1}$ is

(45) $$\frac{\partial^{2}w_{0}}{\partial {T_{0}}^{2}}-I\frac{\partial^{2}{w_{0}}^{\prime \prime }}{\partial {T_{0}}^{2}}+\omega^{2}{w_{0}}^{iv}=0$$

$\varepsilon^{3}$ equation is

(46) $$\frac{\partial^{2}w_{1}}{\partial {T_{0}}^{2}}-I\frac{\partial^{2}{w_{1}}^{\prime \prime }}{\partial {T_{0}}^{2}}+\omega^{2}{w_{1}}^{iv}=-2\frac{\partial^{2}w_{0}}{\partial T_{0}\partial T_{1}}+2I\frac{\partial^{2}{w_{0}}^{\prime \prime }}{\partial T_{0}\partial T_{1}}-\mu_{w}\frac{\partial w_{0}}{\partial T_{0}}+\frac{1}{2}{w_{0}}^{\prime \prime }\int_{0}^{1}{\left({w_{0}}^{\prime }\right)}^{2}dx-Mv^{2}{w_{0}}^{\prime \prime }+qe^{i\Omega \tau }$$

Also let:

(47) $$w_{0}=\sum_{n=1}^{\infty }\phi_{n}(x)\xi_{0n}(\tau ),\;w_{1}=\sum_{n=1}^{\infty }\phi_{n}(x)\xi_{1n}(\tau )$$

Substitute Equation (47) into Equations (45) and (46):

$\varepsilon^{1}$ equation is

(48) $$\sum_{n=1}^{\infty }{\ddot{\xi }}_{0n}\phi_{n}-I\sum_{n=1}^{\infty }{\ddot{\xi }}_{0n}{\phi_{n}}^{\prime \prime }+\omega^{2}\sum_{n=1}^{\infty }\xi_{0n}{\phi_{n}}^{iv}=0$$

$\varepsilon^{3}$ equation is

(49) $$\sum_{n=1}^{\infty }{\ddot{\xi }}_{1n}\phi_{n}-I\sum_{n=1}^{\infty }{\ddot{\xi }}_{1n}{\phi_{n}}^{\prime \prime }+\omega^{2}\sum_{n=1}^{\infty }\xi_{1n}{\phi_{n}}^{iv}=\;-2\frac{\partial^{2}}{\partial T_{0}\partial T_{1}}\sum_{n=1}^{\infty }\xi_{0n}\phi_{n}+2I\frac{\partial^{2}}{\partial T_{0}\partial T_{1}}\sum_{n=1}^{\infty }\xi_{0n}{\phi_{n}}^{\prime \prime }-\mu_{w}\sum_{n=1}^{\infty }{\dot{\xi }}_{0n}\phi_{n}+\frac{1}{2}\sum_{n=1}^{\infty }{\xi_{0n}}^{3}{\phi_{n}}^{\prime \prime }\int_{0}^{1}{\phi_{n}}^{\prime 2}dx-Mv^{2}\sum_{n=1}^{\infty }\xi_{0n}{\phi_{n}}^{\prime \prime }+qe^{i\Omega \tau }$$

where $(\;\dot{)}=d/d\tau$$=d/dT_{0}$. Applying the orthogonal method, we can obtain the dynamic equations as

(50) $${\ddot{\xi }}_{0m}+\frac{\omega^{2}\int_{0}^{1}{\phi_{n}}^{iv}\phi_{m}dx}{\int_{0}^{1}\phi_{n}\phi_{m}dx-I(\int_{0}^{1}{\phi_{n}}^{\prime \prime }\phi_{m}dx)}\xi_{0m}=0$$

(51) $${\ddot{\xi }}_{1m}+\frac{\omega^{2}\int_{0}^{1}{\phi_{n}}^{iv}\phi_{m}dx}{\int_{0}^{1}\phi_{n}\phi_{m}dx-I(\int_{0}^{1}{\phi_{n}}^{\prime \prime }\phi_{m}dx)}\xi_{1m}=-2\frac{\partial }{\partial T_{1}}{\dot{\xi }}_{0m}-\mu_{w}\frac{\int_{0}^{1}\phi_{n}\phi_{m}dx}{\int_{0}^{1}\phi_{n}\phi_{m}dx-I(\int_{0}^{1}{\phi_{n}}^{\prime \prime }\phi_{m}dx)}{\dot{\xi }}_{0m}+\frac{1}{2}\frac{\int_{0}^{1}{\phi_{n}}^{\prime \prime }\phi_{m}dx}{\int_{0}^{1}\phi_{n}\phi_{m}dx-\bar{I}(\int_{0}^{1}{\phi_{n}}^{\prime \prime }\phi_{m}dx)}{\xi_{0m}}^{3}\int_{0}^{1}{\phi_{n}}^{\prime 2}dx\;-Mv^{2}\frac{\int_{0}^{1}{\phi_{n}}^{\prime \prime }\phi_{m}dx}{\int_{0}^{1}\phi_{n}\phi_{m}dx-\bar{I}(\int_{0}^{1}{\phi_{n}}^{\prime \prime }\phi_{m}dx)}\xi_{0m}+qe^{i\Omega \tau }\frac{\int_{0}^{1}\phi_{m}dx}{\int_{0}^{1}\phi_{n}\phi_{m}dx-\bar{I}(\int_{0}^{1}{\phi_{n}}^{\prime \prime }\phi_{m}dx)}$$

The natural frequency $\omega_{nf}$ of this nonlinear system can be obtained from Equation (50):

(52) $$\omega_{m}={\left[\frac{\omega^{2}\int_{0}^{1}{\phi_{n}}^{iv}\phi_{m}dx}{\int_{0}^{1}\phi_{n}\phi_{m}dx-\bar{I}(\int_{0}^{1}{\phi_{n}}^{\prime \prime }\phi_{m}dx)}\right]}^{\frac{1}{2}},m=1,2,3\cdots$$

Assuming the general solution of $\xi_{0m}(\tau )$ in Equation (50) is

(53) $$\xi_{0m}(\tau )=A_{0m}(T_{1},T_{2})e^{-i\varsigma_{m}}e^{i\omega_{m}T_{0}}+{\bar{A}}_{0m}(T_{1},T_{2})e^{i\varsigma_{m}}e^{-i\omega_{m}T_{0}}$$

where $\varsigma_{m}$ is the phase angle, *A_0m_* represents the amplitude of the *m*th mode, and ${\bar{A}}_{0m}$ is the complex conjugate of $A_{0m}$. In order to create the fixed-points plot, we assume σ as the tuned frequency and $1=\omega_{m}+\varepsilon \sigma$, $\varepsilon T_{0}=T_{1}$. The external force is expressed as $q_{m}e^{i\Omega \tau }=q_{m}e^{i(\omega_{m}+\varepsilon \sigma )T_{0}}=q_{m}(e^{i\varepsilon \sigma T_{0}}e^{i\omega_{m}T_{0}})=q_{m}e^{i\sigma T_{1}}e^{i\omega_{m}T_{0}}$. Equation (51) can be expressed as

(54) $${\ddot{\xi }}_{1m}+{\omega_{m}}^{2}\xi_{1m}=\;-2(i\omega_{m}{A^{\prime }}_{0m}e^{-i\varsigma_{m}}e^{i\omega_{m}T_{0}}-i\omega_{m}{{\bar{A}}^{\prime }}_{0m}e^{i\varsigma_{m}}e^{-i\omega_{m}T_{0}}+\omega_{m}{\varsigma^{\prime }}_{m}(A_{0m}e^{-i\varsigma_{m}}e^{i\omega_{m}T_{0}}+{\bar{A}}_{0m}e^{i\varsigma_{m}}e^{-i\omega_{m}T_{0}}))\;-B_{m}(i\omega_{m}A_{0m}e^{-i\varsigma_{m}}e^{i\omega_{m}T_{0}}-i\omega_{m}{\bar{A}}_{0m}e^{i\varsigma_{m}}e^{-i\omega_{m}T_{0}})+C_{m}{\left(A_{0m}e^{-i\varsigma_{m}}e^{i\omega_{m}T_{0}}+{\bar{A}}_{0m}e^{i\varsigma_{m}}e^{-i\omega_{m}T_{0}}\right)}^{3}\;-D_{m}(A_{0m}e^{-i\varsigma_{m}}e^{i\omega_{m}T_{0}}+{\bar{A}}_{0m}e^{i\varsigma_{m}}e^{-i\omega_{m}T_{0}})+E_{m}q_{m}e^{i\sigma T_{1}}e^{i\omega_{m}T_{0}}$$

Please see Appendix B for the definition of the coefficients. When considering the excitation of the first mode (*m* = 1) and selecting all terms containing $e^{i\omega_{1}T_{0}}$, i.e., secular terms, the solvability condition for the first mode can be expressed as

(55) $$-2(i\omega_{1}{A_{1}}^{\prime }e^{-i\varsigma_{1}}+\omega_{1}{\varsigma_{1}}^{\prime }A_{1}e^{-i\varsigma_{1}})-B_{1}(i\omega_{1}A_{1}e^{-i\varsigma_{1}})+C_{1}(3{A_{1}}^{2}{\bar{A}}_{1}e^{-i\varsigma_{1}})-D_{1}(A_{1}e^{-i\varsigma_{1}})+E_{1}q_{1}e^{i\sigma T_{1}}=0$$

Similarly, when considering the excitation of the second mode (*m* = 2) and selecting all terms containing $e^{i\omega_{2}T_{0}}$, i.e., secular terms, the solvability condition for the second mode can be expressed as

(56) $$-2(i\omega_{2}{A_{2}}^{\prime }e^{-i\varsigma_{2}}+\omega_{2}{\varsigma_{2}}^{\prime }A_{2}e^{-i\varsigma_{2}})-B_{2}(i\omega_{2}A_{2}e^{-i\varsigma_{2}})+C_{2}(3{A_{2}}^{2}{\bar{A}}_{2}e^{-i\varsigma_{2}})-D_{2}(A_{2}e^{-i\varsigma_{2}})+E_{2}q_{2}e^{i\sigma T_{1}}=0$$

And the solvability condition for the third mode can be expressed as

(57) $$-2(i\omega_{3}{A_{3}}^{\prime }e^{-i\varsigma_{3}}+\omega_{3}{\varsigma_{3}}^{\prime }A_{3}e^{-i\varsigma_{3}})-B_{3}(i\omega_{3}A_{3}e^{-i\varsigma_{3}})+C_{3}(3{A_{3}}^{2}{\bar{A}}_{3}e^{-i\varsigma_{3}})-D_{3}(A_{3}e^{-i\varsigma_{3}})+E_{3}q_{3}e^{i\sigma T_{1}}=0$$

Then we multiply Equation (55) by $e^{i\varsigma_{1}}$ and divide it into the real part and the imaginary part. Then we can obtain the real part of the first mode:

(58) $$-2(\omega_{1}{\varsigma_{1}}^{\prime }A_{1})+C_{1}(3{A_{1}}^{2}{\bar{A}}_{1})-D_{1}(A_{1})=0$$

the imaginary part of the first mode:

(59) $$-2(\omega_{1}{A_{1}}^{\prime })-B_{1}(\omega_{1}A_{1})=0$$

Similarly, multiplying Equation (56) by $e^{i\varsigma_{2}}$, we can obtain the real part and imaginary part of the second mode. By multiplying Equation (57) by $e^{i\varsigma_{3}}$, we can obtain the real part and imaginary part of the third mode. We consider the case that the first mode was excited and let $\Gamma_{A}=\sigma T_{1}+\varsigma_{1}$, and then Equations (58) and (59) can be rewritten as the real part of the first mode:

(60) $$-2(\omega_{1}{\varsigma_{1}}^{\prime }A_{1})+C_{1}(3{A_{1}}^{2}{\bar{A}}_{1})-D_{1}(A_{1})=-E_{1}q_{1}\cos(\Gamma_{A})$$

the imaginary part of the first mode:

(61) $$-2(\omega_{1}{A_{1}}^{\prime })-B_{1}(\omega_{1}A_{1})=-E_{1}q_{1}\sin(\Gamma_{A})$$

We let ${\Gamma_{A}}^{\prime }=\sigma +{\varsigma^{\prime }}_{1}=0$ and then ${\varsigma^{\prime }}_{1}=-\sigma$. Also let $\frac{\partial A_{1}}{\partial T_{1}}=\frac{\partial A_{2}}{\partial T_{2}}=\frac{\partial A_{3}}{\partial T_{3}}=0$ substitute into the solvability condition, add the squares of the real and imaginary parts of the first mode, Equations (60) and (61), and eliminate the time-related terms $\cos(\Gamma_{A})$ and $\sin(\Gamma_{A})$ to obtain

(62) $${\left\{2(\omega_{1}\sigma A_{1})+C_{1}(3{A_{1}}^{2}{\bar{A}}_{1})-D_{1}(A_{1})\right\}}^{2}+{\left\{-B_{1}(\omega_{1}A_{1})\right\}}^{2}={E_{1}}^{2}{q_{1}}^{2}$$

**Figure 6:**
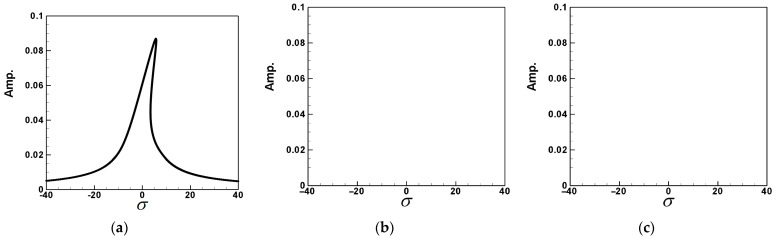
Fixed-points plots for first-mode excitation: (**a**) first mode; (**b**) second mode; (**c**) third mode.

Equation (62) is combined with the real and imaginary parts of the second and third modes to solve for the frequency response of the first mode. The same methodology can be applied to explore scenarios where external forces excite the second and third modes, although a detailed discussion is omitted here. The IMSL’s nonlinear equation solver NEQNF subroutine and Levenberg–Marquardt’s algorithm are employed to solve the simultaneous equations of solvability conditions. Subsequently, the dimensionless amplitudes *A*_1_, *A*_2_, *A*_3_, and the response diagram of the system’s tuning frequency σ are plotted. This enables the generation of fixed-points plots illustrating the impact of external forces on each mode of the system, including the excitation of the first mode (depicted in Figure 6a–c). It is noted that the responses of the second and third modes are zero when the first mode is excited. Although the excitation responses of the second and third modes are not shown due to layout constraints, a summary is provided in Table 1. These plots facilitate the observation of the frequency response of this nonlinear system.

### 3.4. Numerical Analysis of Time Response and Voltage Generation Efficiency

This section aims to validate the accuracy of the fixed-points plots presented in the previous section and analyze the voltage generation effects within this nonlinear system. The fourth-order Runge–Kutta method (RK-4) is employed to solve the problem and generate phase diagrams and time responses. The theoretical power generation voltage is calculated accordingly. Initially, we analyze the equation of the nonlinear fluid–structure coupling system (comprising the flow tube and damper) using the perturbation method. We decompose the term $\xi_{n}$ into the equilibrium periodic term ${\bar{\xi }}_{n}$ and the disturbance periodic term ${\tilde{\xi }}_{n}$. By setting $w=({\bar{\xi }}_{n}(\tau )+{\tilde{\xi }}_{n}(\tau ))\phi_{n}$ and substituting this equality into Equation (21), we obtain

(63) $$({\ddot{\bar{\xi }}}_{n}+{\ddot{\tilde{\xi }}}_{n})\phi_{n}-I({\ddot{\bar{\xi }}}_{n}+{\ddot{\tilde{\xi }}}_{n}){\phi_{n}}^{\prime \prime }+\mu_{w}({\dot{\bar{\xi }}}_{n}+{\dot{\tilde{\xi }}}_{n})\phi_{n}+\omega^{2}({\bar{\xi }}_{n}+{\tilde{\xi }}_{n}){\phi_{n}}^{iv}+\frac{1}{2}M\dot{v}{\left({\bar{\xi }}_{n}+{\tilde{\xi }}_{n}\right)}^{3}{\phi_{n}}^{\prime \prime }\int_{0}^{1}{\phi_{n}}^{\prime 2}dx\;-(\frac{1}{2}+\frac{1}{2}Mv^{2}){\left({\bar{\xi }}_{n}+{\tilde{\xi }}_{n}\right)}^{3}{\phi_{n}}^{\prime \prime }\int_{0}^{1}{\phi_{n}}^{\prime 2}dx+\frac{1}{2}M\dot{v}({\bar{\xi }}_{n}+{\tilde{\xi }}_{n}){\phi_{n}}^{\prime \prime }-M\dot{v}({\bar{\xi }}_{n}+{\tilde{\xi }}_{n}){\phi_{n}}^{\prime \prime }x+\frac{1}{24}Mv^{2}{\left({\bar{\xi }}_{n}+{\tilde{\xi }}_{n}\right)}^{5}{\phi_{n}}^{\prime \prime }\int_{0}^{1}{\phi_{n}}^{\prime 4}dx-\frac{1}{2}M\dot{v}\;{\left({\bar{\xi }}_{n}+{\tilde{\xi }}_{n}\right)}^{3}{\phi_{n}}^{\prime \prime }\int_{0}^{1}\int_{0}^{x}{\phi_{n}}^{\prime 2}dxdx-Mv^{2}{\left({\bar{\xi }}_{n}+{\tilde{\xi }}_{n}\right)}^{3}{\phi_{n}}^{\prime \prime }{\phi_{n}}^{\prime 2}+\frac{5}{8}Mv^{2}{\left({\bar{\xi }}_{n}+{\tilde{\xi }}_{n}\right)}^{5}{\phi_{n}}^{\prime \prime }{\phi_{n}}^{\prime 4}+M\dot{v}({\bar{\xi }}_{n}+{\tilde{\xi }}_{n}){\phi_{n}}^{\prime }\;-\frac{1}{6}M\dot{v}{\left({\bar{\xi }}_{n}+{\tilde{\xi }}_{n}\right)}^{3}{\phi_{n}}^{\prime 3}-Mv^{2}{\left({\bar{\xi }}_{n}+{\tilde{\xi }}_{n}\right)}^{2}({\dot{\bar{\xi }}}_{n}+{\dot{\tilde{\xi }}}_{n}){\phi_{n}}^{\prime }{\phi_{n}}^{\prime \prime }\phi_{n}-\frac{3}{2}Mv{\left({\bar{\xi }}_{n}+{\tilde{\xi }}_{n}\right)}^{2}({\dot{\bar{\xi }}}_{n}+{\dot{\tilde{\xi }}}_{n}){\phi_{n}}^{\prime 3}\;+2Mv({\dot{\bar{\xi }}}_{n}+{\dot{\tilde{\xi }}}_{n}){\phi_{n}}^{\prime }+Mv^{2}({\bar{\xi }}_{n}+{\tilde{\xi }}_{n}){\phi_{n}}^{\prime \prime }-q_{n}e^{i\Omega \tau }-{\omega_{x}}^{2}[({\bar{\xi }}_{n}+{\tilde{\xi }}_{n}){\left.\phi_{n}\right|}_{x=L_{M}}-w_{M}]=0$$

Here, $\phi_{n}{|}_{x=L_{M}}$ represents the displacement of the vibration absorber positioned in the fluid pipeline *L_M_*, and the index *n* signifies each mode, where *n* takes values of 1, 2, 3, and so forth. Multiplying Equation (63) by $\phi_{m}$ for orthogonalization and disregarding higher-order terms ${\xi_{n}}^{4}$ and ${\xi_{n}}^{5}\cdots$, the equation of the nonlinear fluid–structure coupling system (comprising the flow tube and damper) can be simplified to

(64) $$\left({\ddot{\bar{\xi }}}_{n}+{\ddot{\tilde{\xi }}}_{n})+\mu_{w}Q_{1}({\dot{\bar{\xi }}}_{n}+{\dot{\tilde{\xi }}}_{n})+\omega^{2}Q_{2}({\bar{\xi }}_{n}+{\tilde{\xi }}_{n})+\frac{1}{2}M\dot{v}Q_{3}{\left({\bar{\xi }}_{n}+{\tilde{\xi }}_{n}\right)}^{3}-(\frac{1}{2}+\frac{1}{2}Mv^{2})Q_{3}{\left({\bar{\xi }}_{n}+{\tilde{\xi }}_{n}\right)}^{3}+\frac{1}{2}M\dot{v}Q_{4}({\bar{\xi }}_{n}+{\tilde{\xi }}_{n})-M\dot{v}Q_{5}({\bar{\xi }}_{n}+{\tilde{\xi }}_{n}\right)\;+\frac{1}{24}Mv^{2}Q_{6}{\left({\bar{\xi }}_{n}+{\tilde{\xi }}_{n}\right)}^{5}-\frac{1}{2}M\dot{v}Q_{7}{\left({\bar{\xi }}_{n}+{\tilde{\xi }}_{n}\right)}^{3}-Mv^{2}Q_{8}{\left({\bar{\xi }}_{n}+{\tilde{\xi }}_{n}\right)}^{3}+\frac{5}{8}Mv^{2}Q_{9}{\left({\bar{\xi }}_{n}+{\tilde{\xi }}_{n}\right)}^{5}+M\dot{v}Q_{10}({\bar{\xi }}_{n}+{\tilde{\xi }}_{n})-\frac{1}{6}M\dot{v}Q_{11}{\left({\bar{\xi }}_{n}+{\tilde{\xi }}_{n}\right)}^{3}\;-Mv^{2}Q_{12}{\left({\bar{\xi }}_{n}+{\tilde{\xi }}_{n}\right)}^{2}({\dot{\bar{\xi }}}_{n}+{\dot{\tilde{\xi }}}_{n})-\frac{3}{2}MvQ_{11}{\left({\bar{\xi }}_{n}+{\tilde{\xi }}_{n}\right)}^{2}({\dot{\bar{\xi }}}_{n}+{\dot{\tilde{\xi }}}_{n})+2MvQ_{10}({\dot{\bar{\xi }}}_{n}+{\dot{\tilde{\xi }}}_{n})\;+Mv^{2}Q_{4}({\bar{\xi }}_{n}+{\tilde{\xi }}_{n})-q_{n}e^{i\Omega \tau }Q_{13}-{\omega_{x}}^{2}[Q_{14}\xi_{n}-Q_{15}w_{M}]=0$$

The parameters of each coefficient are marked in Appendix B. Next, we analyze the equation of the vibration absorber, including the repulsion force (*F_M_*) of the magnets; orthogonalizing Equation (26), we can obtain

(65) $${\ddot{w}}_{M}-{\omega_{x}}^{2}(Q_{16}\xi_{n}-w_{M})-\frac{\mu_{M}M_{A}V_{A}M_{B}V_{B}}{4\pi }Q_{17}{\left[Q_{17}\xi_{S}+w_{M}+\bar{d}+{\left(2Q_{17}w_{M}\xi_{S}\right)}^{1/2}\right]}^{-7}(3{Q_{17}}^{3}{\xi_{S}}^{3}+9{Q_{17}}^{2}w_{M}{\xi_{S}}^{2}\;+12{Q_{17}}^{2}{w_{M}}^{2}\bar{d}+9Q_{17}{w_{M}}^{2}\xi_{S}+24Q_{17}w_{M}\xi_{S}\bar{d}-4Q_{17}\xi_{S}{\bar{d}}^{2}+3{w_{M}}^{3}+12{w_{M}}^{2}\bar{d}-4w_{M}{\bar{d}}^{2}+3{\bar{d}}^{3})=0$$

Equation (65) represents the equation of the vibration absorber, incorporating the coupling magnet repulsion (Lorentz force). The parameters for each coefficient are annotated in Appendix B. Finally, employing the perturbation method in conjunction with Equation (30), we orthogonalize the equation to obtain

(66) $$\left({\ddot{\bar{\xi }}}_{S}+{\ddot{\tilde{\xi }}}_{S})+\mu_{b}({\dot{\bar{\xi }}}_{S}+{\dot{\tilde{\xi }}}_{S})+({\bar{\xi }}_{S}+{\tilde{\xi }}_{S})Q_{18}+\frac{1}{Q_{19}}[Q_{20}{\left({\bar{\xi }}_{S}+{\tilde{\xi }}_{S}\right)}^{3}+Q_{21}{\left({\bar{\xi }}_{S}+{\tilde{\xi }}_{S}\right)}^{3}+4Q_{22}{\left({\bar{\xi }}_{S}+{\tilde{\xi }}_{S}\right)}^{3}\right]\;-\frac{1}{Q_{19}}\frac{\hat{k}\eta^{2}}{e^{R\tau }}\left[Q_{23}({\bar{\xi }}_{S}+{\tilde{\xi }}_{S})\right.\left.\left({\dot{\bar{\xi }}}_{S}+{\dot{\tilde{\xi }}}_{S}\right)\right]+\frac{1}{2Q_{19}}\left[\left({\bar{\xi }}_{S}+{\tilde{\xi }}_{S})(({\ddot{\bar{\xi }}}_{S}+{\ddot{\tilde{\xi }}}_{S})({\bar{\xi }}_{S}+{\tilde{\xi }}_{S}\right)\right.+2{\left({\dot{\bar{\xi }}}_{S}+{\dot{\tilde{\xi }}}_{S}\right)}^{2}+({\bar{\xi }}_{S}+{\tilde{\xi }}_{S})({\ddot{\bar{\xi }}}_{S}+{\ddot{\tilde{\xi }}}_{S}))\left.Q_{24}\right]\;+\frac{\mu_{M}M_{A}V_{A}M_{B}V_{B}}{4\pi }\left\{\frac{5Q_{17}[{\left(w_{M}+Q_{17}(\xi_{S})\right)}^{3}+3\bar{d}{\left(w_{M}+Q_{17}(\xi_{S})\right)}^{2}-2{\bar{d}}^{2}(w_{M}+Q_{17}(\xi_{S}))]}{{\left[{\left(w_{M}+Q_{17}(\xi_{S})\right)}^{2}+{\bar{d}}^{2}\right]}^{7/2}}\right.-\left.\frac{3\bar{d}Q_{17}+2Q_{17}(w_{M}+Q_{17}(\xi_{S}))}{{\left[{\left(w_{M}+Q_{17}(\xi_{S})\right)}^{2}+{\bar{d}}^{2}\right]}^{5/2}}\right\}=0$$

**Figure 7:**
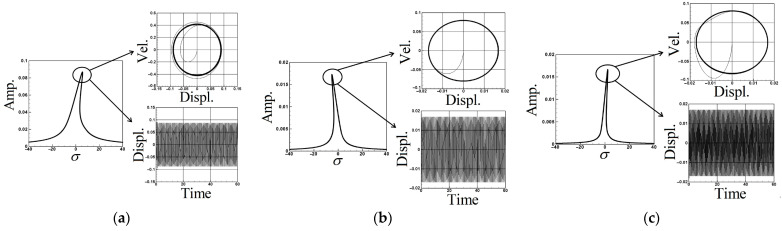
Validation of fixed-points plot, phase plot, and time response: (**a**) first mode; (**b**) second mode; (**c**) third mode.

The parameters for each coefficient are provided in Appendix B. We substitute the first, second, and third modes into Equations (64)–(66), respectively, and employ the fourth-order Runge–Kutta method (RK-4) to solve the simultaneous solution of the perturbation equation. This enables the solution of time response diagrams for the fluid delivery pipe, vibration absorber, and cantilever beam. Through these diagrams, we determine the amplitude of the additional damper in the flow tube, presenting a numerical closed-loop solution. Utilizing the fixed-points plots from the previous section to determine the maximum amplitude of each mode, we substitute these values into the voltage function (Equation (34)) of the piezoelectric equation. After the calculations, the theoretical voltage for each mode is obtained, and a response diagram of voltage and time is generated. The purpose is to analyze whether the pipeline vibration amplitude obtained using the method of multiple scales (MOMS) is accurate for the VEH system before considering the energy conversion efficiency. Therefore, we compared the fixed-points plots with the amplitudes obtained by the numerical methods. Figure 7a–c provide an interactive verification comparison for the fixed-points plots, phase plots, and time response charts of the first to third modes. As can be seen, the first mode of the system is of the hardening type; therefore, nonlinear geometry has a greater effect on the amplitudes in the system compared to nonlinear inertia. In contrast, the second mode is of the softening type; however, nonlinear geometry has a greater influence on the amplitudes than does nonlinear inertia; therefore, the fixed-points plot of the second mode when the second mode is excited presents a curve toward the right. The first mode of the system exhibits a hardening behavior, indicating that nonlinear geometry has a more significant impact on the amplitudes than nonlinear inertia. Conversely, the second mode demonstrates a softening behavior due to the greater influence of the nonlinear inertia. As a result, the fixed-points plot for the second mode, when excited, curves to the right. Nayfeh and Pai [11] also explained this phenomenon. The results are summarized in Table 2. These confirm the correctness of the fixed-points plots in this study and confirm the amplitude of each mode.

**Figure 8:**
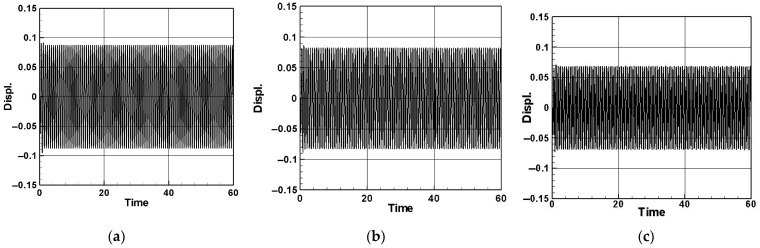
Time response of the tube in the first mode: (**a**) without damper; (**b**) DVA placed at *x* = 0.25; (**c**) DVA placed at *x* = 0.5.

Figure 8 illustrates a comparative analysis of the time response for the system with and without additional vibration absorbers positioned at *x* = 0.25 and *x* = 0.5, using the first mode as an example. The results for the second and third modes are presented in Table 3. The comparisons indicate that introducing a damper notably reduces the displacement and enhances the system stability. Particularly noteworthy is the pronounced vibration reduction effect observed when the damper is positioned at the midpoint of the pipe (*x* = 0.5), resulting in a substantial decrease in the pipeline vibration displacement and stable system convergence.

**Figure 9:**
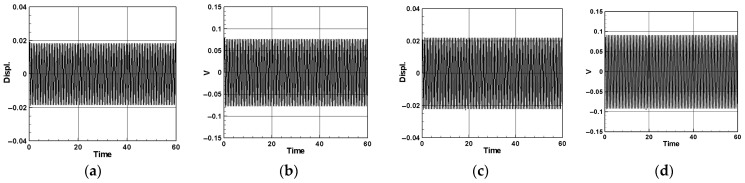
Time responses and dimensionless voltage values of the beam in the first mode: (**a**) time response with the DVA placed at *x* = 0.25; (**b**) dimensionless voltage with the DVA placed at *x* = 0.25; (**c**) time response with the DVA placed at *x* = 0.5; (**d**) dimensionless voltage with the DVA placed at *x* = 0.5.

Figure 9 illustrates the scenario where the mass of the vibration absorber is set to 5% of the mass of the flow tube, positioned at *x* = 0.25 and *x* = 0.5 along the flow tube. Using the first mode as an example, the time response of the elastic cantilever beam and the dimensionless voltage diagram of this MDDI VEH system are presented. The results for the second and third modes are detailed in Table 4 and Table 5. A key observation from these comparisons is that, when the system damper is centrally located (*x* = 0.5), the elastic cantilever beam exhibits a larger displacement. Furthermore, by comparing the dimensionless voltage diagrams, it is evident that significant displacement of the elastic cantilever beam results in the concurrent deformation of the PZT, leading to an increase in the generated voltage. Specifically, when the vibration absorber is positioned at *x* = 0.5, the generated voltage is significantly greater than when the vibration absorber is located at *x* = 0.25. This implies that, when the vibration absorber is centrally placed, it achieves more effective vibration reduction and yields a larger dimensionless theoretical voltage. 

## 4. Experimental Analysis

### 4.1. Experimental Set Up

**Figure 10:**
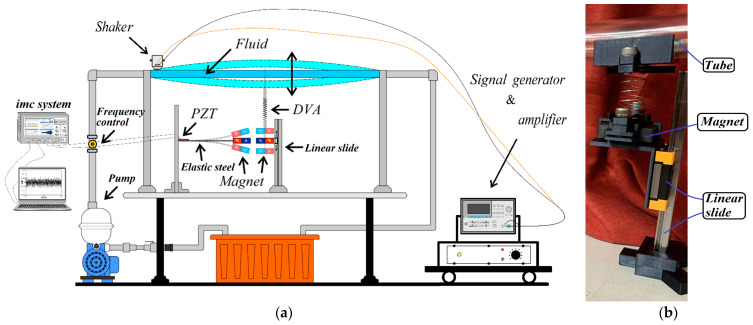
Flow tube damping and magnetoelectric MDDI vibration energy harvesting system: (**a**) experimental schematic diagram; (**b**) linear slide and vibration absorber.

**Figure 11:**
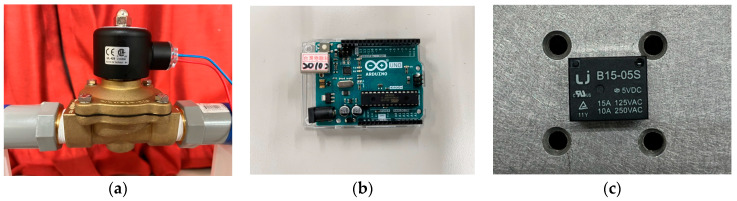
Flow tube flow field frequency control components: (**a**) solenoid valve; (**b**) Arduino UNO; (**c**) relay.

To further validate the feasibility of this study, we construct a simple experiment based on the theoretical model depicted in Figure 1. The energy harvesting mechanism comprises three key components: the flow tube, the DVA (including a magnet), and the elastic cantilever beam with a PZT. The cantilever beam (elastic steel) has dimensions of 110 × 31 × 0.074 mm, with a Young’s modulus of 200 × 10^9^ N/m². The piezoelectric patch is composed of PZT-grade PZT-5H, with dimensions of 60 × 20 × 0.075 mm. The large magnet measures 3.9 × 2.4 × 0.9 cm, while the small magnet measures 4.8 × 2.8 × 0.2 cm. The design principle of the energy harvesting system involves installing a damper in the vibrating flow tube to absorb the vibrations of the flow tube. The damper’s vibrations, combined with the repulsive force of the magnet, drive the elastic steel with the PZT to vibrate. This vibration allows the PZT to convert mechanical energy into electrical energy. In general, if the vibration absorber can effectively dampen most of the vibrations in the main body, the main body can achieve vibration reduction, but the vibration absorber itself will experience greater vibrations. To optimize the system, proper installation of the vibration absorber at the optimal position along the flow tube is crucial. This ensures effective damping of the main body’s vibrations and allows the vibration absorber to provide optimal electric power generation. In the experiment, a water pump (Figure 10a) is utilized to introduce water flow into the flow tube. A solenoid valve (Figure 11a) is positioned in front of the water inlet of the tube. An Arduino UNO board (Figure 11b) is employed to output a digital signal to the relay (Figure 11c), causing on-and-off circuit states. This design establishes a straightforward valve control circuit switch system. A solderless breadboard is then used to interconnect the system and power supply to the solenoid valve, enabling the control of the water flow delivered by the pump so that the fluid in the tube flows at a specific speed. In this experiment, the flow speed is fixed at 0.425 m/s. Fixing the flow speed simplifies the mathematical modeling and experimental setup, making it easier to validate theoretical predictions against experimental data. Using a fixed flow speed also allows us to maintain consistent experimental conditions. This consistency is crucial for accurately isolating and evaluating the performance of the magneto-electric flow-coupled MDDI VEH system. By eliminating flow speed as a variable, we can ensure that observed effects and results are directly attributable to the system’s design and functionality rather than fluctuations in flow speed. 

**Figure 12:**
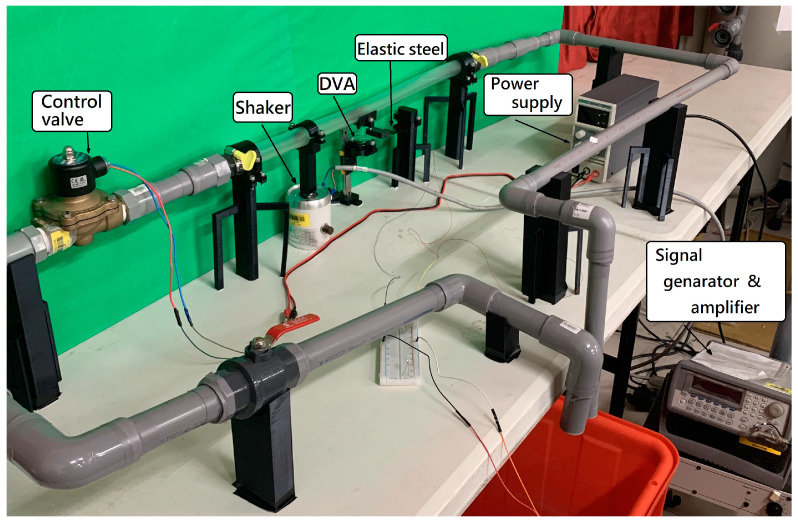
Experimental setup diagram.

Additionally, a shaker is installed to excite specific positions of the flow tube to simulate the situation when the pipeline is excited by different external forces. The purpose of using both a shaker and the controlled water flow system is to simulate different types of excitations that the system could encounter in real-world scenarios. The shaker provides controlled mechanical excitation at specific frequencies and amplitudes, effectively simulating external vibrational forces. While the water flow system induces fluid–structure interaction at a constant speed, the shaker adds a layer of dynamic excitation that represents environmental vibrations and operational machinery forces. This combined approach ensures that the system’s performance can be evaluated under a broader range of conditions, thereby enhancing the prediction of the system’s behavior in practical applications where pipelines may be subjected to both internal fluid dynamics and external forces. A vibration absorber is strategically placed under the flow tube, with a magnet attached to the absorber’s mass. This magnet is affixed to a linear slide (Figure 10b), allowing it to vibrate in an up-and-down motion. Positioned adjacent to it is an elastic steel beam, anchored at its root to a base, with a piezoelectric patch attached. At the free end of the beam, a magnet with the same polarity as the magnet on the vibration absorber is placed, forming an MDDI vibration energy harvesting system (Figure 10a). Combining all these components completes the assembly of a simple experimental instrument device. The final experimental setup is illustrated in Figure 12.

### 4.2. Measurement of the Natural Frequencies

**Figure 13:**
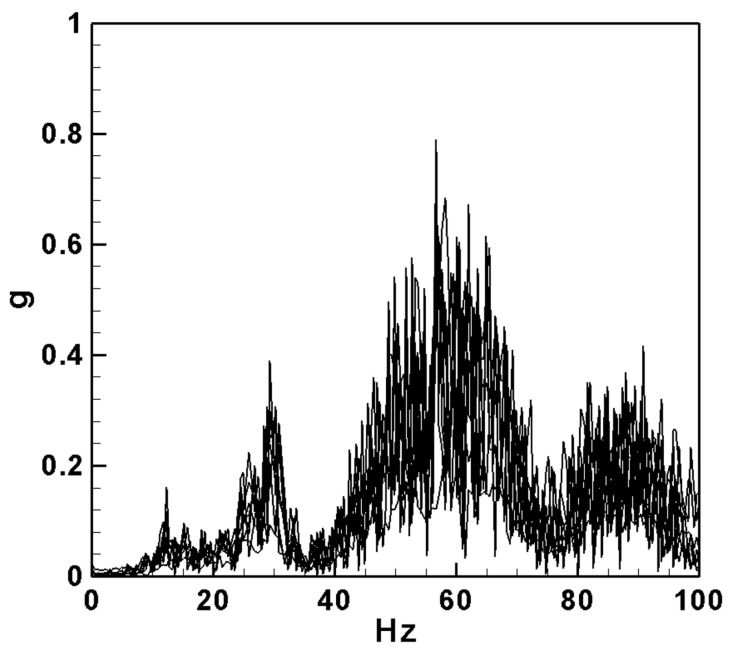
Frequency Response Function (FRF) showing peaks at the natural frequencies.

Initially, the natural frequency of the pipe is determined through measurement. An impact hammer (Meggitt, Coventry, UK, model 2302 impact hammer) and an accelerometer are employed to measure the voltage using the imc^TM^ system (CS-5008-1, TÜV Rheinland, Kölle, Germany). Accelerometers are mounted at the 1/4, 1/2, and 3/4 positions along the pipe. An impact hammer excites the pipe at these same positions, with 3–5 strikes performed at each location. The resulting data are analyzed to determine the natural frequencies. Subsequently, fast Fourier transform is applied to obtain the natural frequency. These experimentally obtained frequency values will be compared with the theoretical analysis frequency values ($\omega_{m}={\left[\frac{\omega^{2}\int_{0}^{1}{\phi_{n}}^{iv}\phi_{m}dx}{\int_{0}^{1}\phi_{n}\phi_{m}dx-\bar{I}(\int_{0}^{1}{\phi_{n}}^{\prime \prime }\phi_{m}dx)}\right]}^{\frac{1}{2}}$, *m* = 1, 2, 3...) to validate the accuracy of the theoretical predictions. After measurement using imc^©^, the natural frequencies of the system are obtained and shown in Figure 13. The first-mode frequency is 12.210 Hz, the second mode is 29.291 Hz, and the third mode is 56.641 Hz. The theoretical predictions of the natural frequencies, calculated using Equation (52), are 12.886 Hz for the first mode, 30.652 Hz for the second mode, and 57.471 Hz for the third mode. These results are summarized in Table 6 below.

From Table 6, we can compare the theoretical and experimental natural frequencies of the first three modes of the system. Since the frequency of the third mode is quite high and has a minimal impact on the power generation efficiency of the system, we will focus on 12.210 Hz and 29.291 Hz as the excitation frequencies of the first two modes in the subsequent experiments.

### 4.3. Internal Resistance Measurement

Prior to delving into the analysis of the electric power in this fluid-structure coupled magneto-electric energy harvesting system, an additional resistor needs to be introduced into the system as a load. This step is essential for acquiring the electric power generated by the system. To optimize the electric power output, it is advantageous to set the additional resistance value as close as possible to the internal resistance value of the system. The electric power equation is formulated as follows:

(67) $$P=\bar{I}\bar{V}={\bar{I}}^{2}R=\frac{{\bar{V}}^{2}}{R}$$

where *P* is electric power, $\bar{I}$ is current, and $\bar{V}$ is voltage. Use Thevenin’s Theorem to obtain the internal resistance of the system, and its equation is:

(68) $${\bar{V}}_{L}=\frac{R_{L}}{R_{T}+R_{L}}{\bar{V}}_{T}$$

where $R_{T}$ is the internal resistance, ${\bar{V}}_{L}$ is the load voltage, ${\bar{V}}_{T}$ is the open circuit voltage, and $R_{L}$ is the load resistance, which can be combined into the following equation by Equations (67) and (68):

(69) $$P=\frac{{\bar{V}}_{L}^{2}}{R_{L}}=\frac{\frac{R_{L}^{2}}{{\left(R_{T}+R_{L}\right)}^{2}}{\bar{V}}_{T}^{2}}{R_{L}}=\frac{R_{L}{\bar{V}}_{T}^{2}}{{\left(R_{T}-R_{L}\right)}^{2}+4R_{T}R_{L}}=\frac{{\bar{V}}_{T}^{2}}{\frac{{\left(R_{T}-R_{L}\right)}^{2}}{R_{L}}+4R_{T}}$$

First, we used a 430-ohm resistor as the load resistor for the system and measured the system’s average voltage. Since the mass of the DVA affects the internal resistance value of the system, we analyzed the internal resistance values of the system with two different masses of DVAs. We used magnets with masses of 45 g (large magnet) and 35 g (small magnet) as the mass for the DVA. Their volumes are 8.424 and 2.688 cm³, respectively. The magnets installed at the free end of the elastic steel are identical to those used in the DVA. Through measurements with imc©, the open-circuit average voltage of the system with the small-magnet damper was 1.56847 volts. When a 430-ohm resistor was connected in series, the average load voltage of the system was 0.01325 volts. Substituting this into Equation (68), we obtained the theoretical internal resistance value to be 50.471 K ohms.

**Figure 14:**
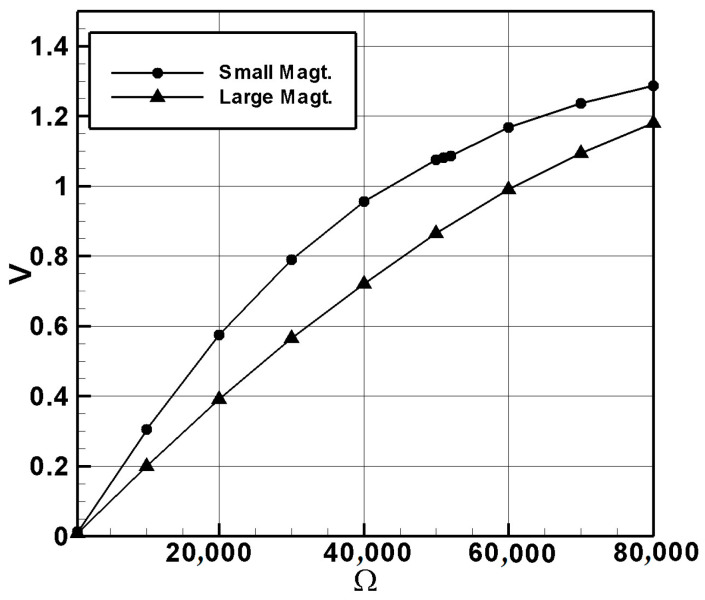
Ohm–voltage plot for the small/large-magnet system.

**Figure 15:**
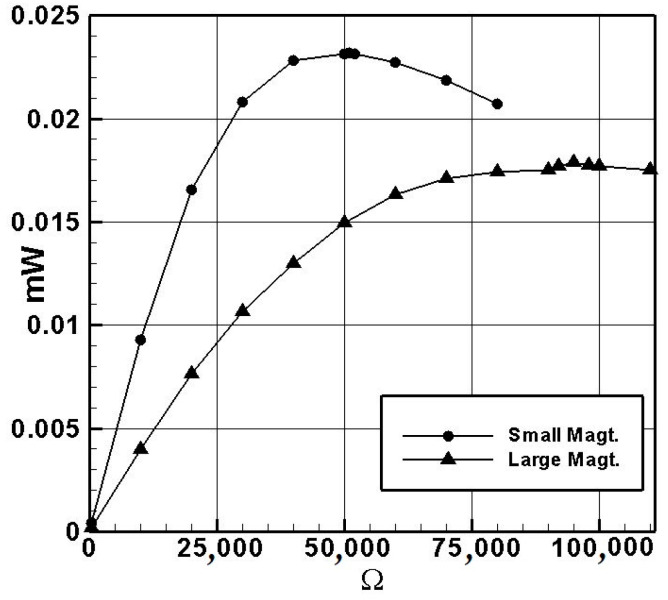
Ohm-power plot for the small/large-magnet system.

Next, we chose other resistors of different ohms for the system, measured their average voltage, and plotted the ohm–voltage graph (Figure 14) and the ohm–power graph (Figure 15) for the small- and large-magnet systems. The results are summarized in Table 7 and Table 8. In Table 7, A–E represent the average voltages from the five experiments. From the ohm–power graph (Figure 15) for the small-magnet system, it can be seen that, when the resistance is 50.45 K ohms, the small-magnet system has the maximum output power. Following the same procedure, we measured the internal resistance of the system induced by the large-magnet DVA. The open-circuit average voltage was 1.88958 volts. When a 430-ohm resistor was connected in series, the measured average voltage was 0.00846 volts. The theoretical internal resistance value was found to be 95.612 K ohms. Similarly, using resistors of different ohms for the system, we plotted the ohm–voltage graph (Figure 14) and the ohm–power graph (Figure 15) for the large-magnet system. From Figure 15, it can be seen that, when the load resistance is 95 K ohms, the large-magnet system has the maximum output power. These two resistance values will be used as the load resistance values in subsequent experiments to ensure the experiments yield the maximum output power.

### 4.4. System Displacement Measurement and Theoretical Verification

**Figure 16:**
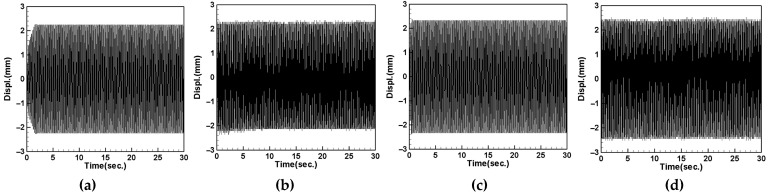
Displacement of the first mode: (**a**) theoretical displacement diagram with the shaker placed at 1/4 of the tube length; (**b**) experimental displacement diagram with the shaker placed at 1/4 of the tube length; (**c**) theoretical displacement diagram with the shaker placed at 1/2 of the tube length; (**d**) experimental displacement diagram with the shaker placed at 1/2 of the tube length.

This section presents the experimental measurement of the vibration reduction effect of the DVA attached to the flow tube. Firstly, we used a shaker to simulate the vibration conditions of the flow tube and positioned it at 1/4, 1/2, and 3/4 of the length of the flow tube. Using a laser displacement gauge (LDG), we measured the displacement of the first and second modes of the flow tube and compared it with the theoretical displacement (due to space constraints, only the first-mode pipe amplitude is illustrated in Figure 16). After calculating the root mean square, the results of all the combinations are summarized in Table 9.

Table 9 shows the theoretical displacement values, experimental displacement values, and errors for the first mode (12.21 Hz) and second mode (29.291 Hz) when the vibrator is positioned at different locations. From the table, the displacement error between the theoretical values and the experimental values is within 5%, indicating that the theoretical predictions are highly accurate.

#### 4.4.1. Tube Vibration with DVA at Midpoint

**Figure 17:**
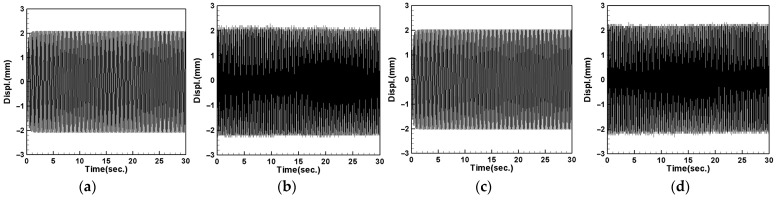
Displacement of the first mode of the system with a large-magnet DVA placed at 1/2 of the tube length: (**a**) theoretical displacement diagram with the shaker placed at 1/4 of the tube length; (**b**) experimental displacement diagram with the shaker placed at 1/4 of the tube length; (**c**) theoretical displacement diagram with the shaker placed at 1/2 of the tube length; (**d**) experimental displacement diagram with the shaker placed at 1/2 of the tube length.

We placed a vibration absorber at the midpoint (1/2) of the flow tube, using the large magnet as the mass of the DVA system. The results are then compared with the theoretical values (the first mode flow tube vibration amplitude is shown in Figure 17). After organizing all the combinations, the results are presented in Table 10 and Table 11.

Table 10 compares the theoretical and experimental root mean square values of the tube with a large-magnet DVA positioned at 1/2 tube length, displaying the theoretical and experimental damping effects and vibration amplitudes. When the DVA is placed at the midpoint of the tube, the damping effect is optimal. When the shaker is placed at 1/4 and 3/4 of the tube, the displacement values are nearly equal, and the differences between the theoretical and experimental values are all less than 5%. 

Next, we replace the mass of the DVA with a small magnet and conduct the same experiments. We measure the displacement values of the tube when the DVA is placed at 1/2 tube length with the shaker positioned at three different locations along the flow tube and compare the results with the theoretical values. After calculating the root mean square, the results are organized in Table 11.

Table 11 presents a comparison of the theoretical and experimental root mean square values of the system with a small magnet DVA. From the tables, it can be observed that placing the DVA at the midpoint of the tube results in optimal damping effects for both the first and second modes. Additionally, the discrepancies between the theoretical and experimental values are within 5%, indicating a high level of accuracy in the theoretical predictions. Comparing Table 10 and Table 11, it can be concluded that placing the shaker and small-magnet DVA at the midpoint of the flow tube yields the best damping effects. 

#### 4.4.2. Tube Vibration with DVA at 1/4 Tube Length

We moved the DVA to the 1/4 position to observe and measure its effect on the tube’s vibration damping by changing its placement. We compared the results with the theory, and after taking the root mean square and organizing the data, Table 12 was obtained. 

From Table 12, it can be observed that when the DVA and shaker are both positioned at 1/4 of the tube length, the maximum damping effect occurs. Following this, when the shaker is positioned at 1/2 and 3/4 of the tube length, the damping effect decreases. Therefore, it is inferred that, the closer the proximity between the vibration source and the DVA, the better the damping effect; conversely, the farther apart the positions of the vibration source and DVA, the poorer the damping effect. Continuing with the above results, the DVA system with the large magnet is replaced with a DVA system with a small magnet. A comparison with the theoretical values is completed to observe if the same phenomenon occurs, and the results are organized into Table 13 after taking the root mean square.

From Table 13, it is evident that when both the DVA and shaker are positioned at 1/4 of the tube length, the flow tube exhibits the most significant damping effect. This result is consistent with the large-magnet system. Additionally, based on the results from Table 10, Table 11, Table 12 and Table 13, it is found that, when both the vibration source and DVA are located at 1/2 of the tube length, the optimal damping of the pipeline is achieved. Moreover, the damping amplitude of the small-magnet vibration absorber surpasses that of the large-magnet vibration absorber system.

#### 4.4.3. Tube Displacement with Different DVA Spring Constants

Two additional springs with different elastic coefficients with k = 13.3 and 12.5 were selected for comparison with the spring with k = 20, and experiments were conducted for both the large- and small-magnet DVA systems. Since the experiments on the placement of the shaker and DVA have already been performed as described above, we selected combinations where both the shaker and DVA are positioned at 1/2 of the tube length for comparison. By using the RK-4 numerical method and comparing the results with the experimental data, Table 14 and Table 15 were compiled.

From Table 14 and Table 15, it is evident that the spring constant of the spring significantly affects the damping effect. When the spring constant is k = 20, both the large-magnet DVA system and the smal-magnet DVA system exhibit better damping effects, particularly noticeable in the small-magnet system. For the first mode, the theoretical and experimental damping amplitudes are 19% and 18%, respectively. For the second mode, the theoretical and experimental damping amplitudes are 50% and 47%, respectively. These damping effects are superior to those observed with spring constants k = 13.3 and 12.2. Based on all the experiments conducted, we conclude that positioning the DVA at 1/2 of the tube length results in the optimal damping effect, and the small-magnet DVA system is more suitable than the large-magnet system. Furthermore, choosing a spring constant of k = 20 for the spring provides the best damping combination.

### 4.5. Voltage Measurement and Theoretical Verification of the System

In this section, we will discuss the output voltage of the system. The output voltage is obtained by measuring the piezoelectric patch (PZT) located at the root of the cantilever beam using the imc© device. Additionally, we obtained the theoretical voltage equation for the system in the previous sections. By substituting different parameters such as the mass of the magnet and the spring constant of the DVA into Equations (64)–(66) and using the RK-4 numerical method combined with Equation (34), we can derive the theoretical voltage of the system. We will compare the experimental and theoretical output voltages of the system resulting from different DVA masses, positions, and spring constants. The results will be tabulated and compared with the theoretical values to validate the feasibility of the model proposed in this study.

#### 4.5.1. Voltage with DVA Positioned at 1/2 of the Tube Length

**Figure 18:**
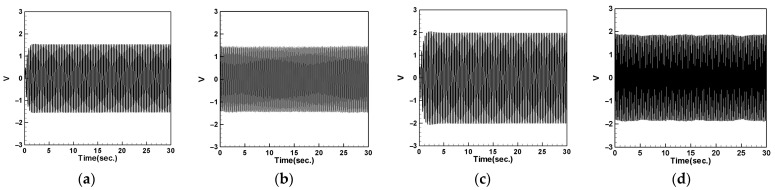
Voltage diagram of the first mode excitation for the system with a large magnet DVA placed at 1/2 of the tube length: (**a**) theoretical voltage diagram with the shaker placed at 1/4 of the tube length; (**b**) experimental voltage diagram with the shaker placed at 1/4 of the tube length; (**c**) theoretical voltage diagram with the shaker placed at 1/2 of the tube length; (**d**) experimental voltage diagram with the shaker placed at 1/2 of the tube length.

**Figure 19:**
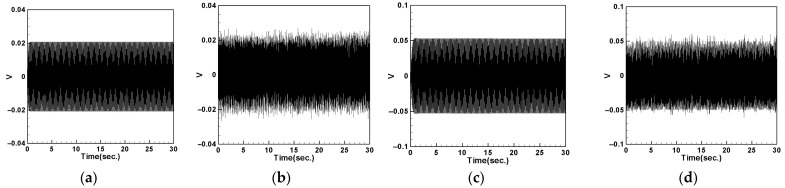
Voltage diagram of the second mode excitation for the system with a large magnet DVA placed at 1/2 of the tube length: (**a**) theoretical voltage diagram with the shaker placed at 1/4 of the tube length; (**b**) experimental voltage diagram with the shaker placed at 1/4 of the tube length; (**c**) theoretical voltage diagram with the shaker placed at 1/2 of the tube length; (**d**) experimental voltage diagram with the shaker placed at 1/2 of the tube length.

First, we place the DVA at 1/2 of the tube length and measure the induced voltages of the first and second modes for both the large- and small-magnet systems. We connect resistors of 50.45 kΩ and 95 kΩ to the small- and large-magnet systems, respectively, to ensure that the system can output the maximum electrical power, and then compare it with the theoretical voltage. Figure 18 shows the voltage diagram of the tube for exciting the first mode with a large-magnet DVA attached at 1/2 of the tube length, where (a) represents the theoretical voltage diagram with the shaker placed at 1/4 of the tube length, (b) represents the experimental voltage diagram with the shaker placed at 1/4 of the tube length, (c) represents the theoretical voltage diagram with the shaker placed at 1/2 of the tube length, and (d) represents the experimental voltage diagram with the shaker placed at 1/2 of the tube length. Similarly, Figure 19 depicts the voltage diagram of the tube for exciting the second mode with a large-magnet DVA attached at 1/2 of the tube length, following the same notation as Figure 18. Due to space limitations, the theoretical and experimental voltage diagrams of the tube’s vibration output for the small-magnet DVA system will be presented in tabular form.

In this study, we varied the excitation frequency (1st and 2nd mode) applied to the system. By applying different frequencies, we explored the system’s behavior under various dynamic conditions and identified the frequencies that are most effective at inducing the desired behaviors. Varying these excitation levels allowed us to assess their impact on the energy harvesting efficiency and overall system stability. This provided a deeper understanding of the underlying dynamics and helped to validate the proposed model. Table 16 presents the root mean square (RMS) values of the theoretical and experimental voltages. Compared to the displacement reduction results in Table 14 and Table 15, the second mode appears to have a better damping effect on the pipe vibration. However, it generates less voltage than the first mode due to the smaller vibration amplitude. According to Table 16, the error between the theoretical and experimental values is approximately 3% for the first mode and about 8% for the second mode. We attribute this discrepancy to the rapid vibration of the elastic steel and the inability of the magnetic repulsive force during the experiment to fully match the recovery force of the elastic steel. Nevertheless, with experimental errors all below 10%, the results of this experiment are sufficient to demonstrate the accuracy of the theoretical voltage predictions. Additionally, based on the results in the tables, it can be observed that, when the shaker is placed at 1/2 of the tube length and the DVA is positioned at the same location, both systems exhibit the maximum output voltage for the first and second modes. Since the DVA achieves optimal damping effects when its position matches that of the shaker, and due to the vigorous vibration of the DVA, the neighboring elastic steel is excited by the repulsive force of the magnets, resulting in greater displacement. Consequently, the PZT located at the root of the elastic steel outputs a larger voltage.

#### 4.5.2. Voltage with DVA Positioned at 1/4 of the Tube Length

**Figure 20:**
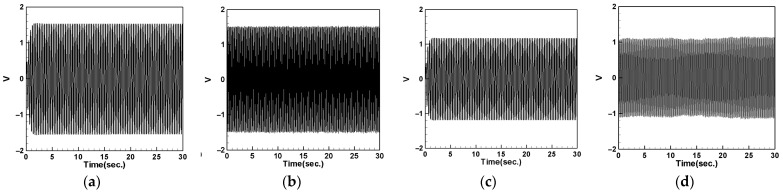
Voltage diagram of the first mode excitation for the system with a large magnet DVA placed at 1/4 of the tube length: (**a**) theoretical voltage diagram with the shaker placed at 1/4 of the tube length; (**b**) experimental voltage diagram with the shaker placed at 1/4 of the tube length; (**c**) theoretical voltage diagram with the shaker placed at 1/2 of the tube length; (**d**) experimental voltage diagram with the shaker placed at 1/2 of the tube length.

**Figure 21:**
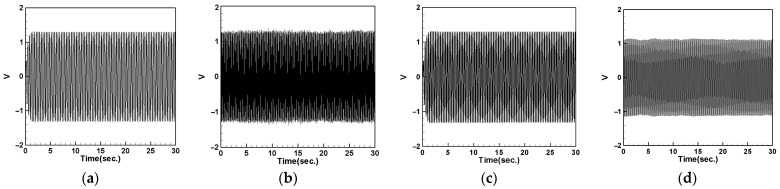
Voltage diagram of the first mode excitation for the system with a small magnet DVA placed at 1/4 of the tube length: (**a**) theoretical voltage diagram with the shaker placed at 1/4 of the tube length; (**b**) experimental voltage diagram with the shaker placed at 1/4 of the tube length; (**c**) theoretical voltage diagram with the shaker placed at 1/2 of the tube length; (**d**) experimental voltage diagram with the shaker placed at 1/2 of the tube length.

Next, we change the position of the DVA, placing it at 1/4 of the tube length, and measure the output voltage of the system using the same method. Comparing the theoretical and experimental voltage for the first mode (Figure 20 and Figure 21), it can be observed that both the large- and small-magnet systems exhibit optimal output voltage when the DVA is positioned at 1/4 of the tube length. Due to space limitations, the theoretical and experimental voltage diagrams of the tube’s vibration output for the second mode will be presented in tabular form, compiled into Table 17.

Table 17 reveals that, when both the shaker and DVA are positioned at 1/4 of the tube length, both the large- and small-magnet systems exhibit the maximum theoretical and experimental voltages. As the distance between the vibration source and DVA increases, both the theoretical and experimental voltages decrease accordingly. Compared with Table 16, it can be noted that the maximum output voltage occurs when the shaker and DVA are placed at the same location, and the output voltage is greater when the DVA is positioned at 1/2 of the tube length compared to when it is positioned at 1/4 of the tube length. Therefore, we can confirm that, when the DVA is located at 1/2 of the tube length and the shaker is at the same position, the system exhibits the maximum output voltage. Subsequent experiments will adopt this configuration as the basis and observe the effect of changing the spring constant.

#### 4.5.3. System Output Voltage with Different DVA Spring Constants

**Figure 22:**
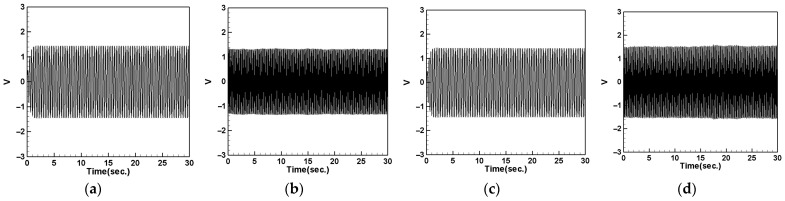
Voltage diagram of the first-mode excitation for the system with a large-magnet damper: (**a**) theoretical voltage diagram with spring constant k = 13.3; (**b**) experimental voltage diagram with spring constant k = 13.3; (**c**) theoretical voltage diagram with spring constant k = 12.5; (**d**) experimental voltage diagram with spring constant k = 12.5.

**Figure 23:**
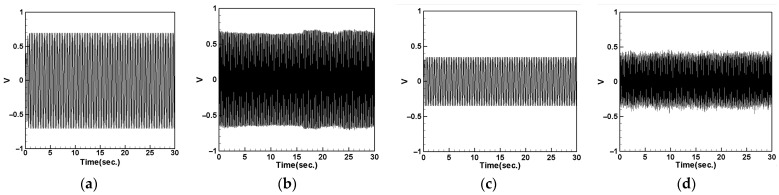
Voltage diagram of the first-mode excitation for the system with a small-magnet DVA: (**a**) theoretical voltage diagram with spring constant k = 13.3; (**b**) experimental voltage diagram with spring constant k = 13.3; (**c**) theoretical voltage diagram with spring constant k = 12.5; (**d**) experimental voltage diagram with spring constant k = 12.5.

This section analyzes two additional spring constants, k = 13.3 and 12.5, and compares them with the previously used spring constant, k = 20. The theoretical and experimental output voltages are depicted in Figure 22 and Figure 23. The RMS voltages for each combination are listed in Table 18.

In Table 18, when the spring constant changes from k = 20 to k = 13.3, the experimental output voltage for the first mode decreases from 1.30340 volts to 0.94948 volts, and further decreases to 0.94088 volts when using k = 12.5. This indicates that the spring constant k = 20 provides better output voltage for the large-magnet system. Table 18 shows that spring constant k = 20 also yields superior electrical generation performance for the small-magnet system. The error between the theoretical and experimental voltages for these two systems is approximately 3% for the first mode and around 8% for the second mode. The cause of this discrepancy is consistent with the explanation provided earlier, attributed to the rapid vibration of the high-mode DVA and the mismatch between the repulsive force of the magnets and the recovery force of the elastic steel, resulting in a higher error in the second-mode vibration frequency compared to the first mode. However, with errors below 10%, these results still sufficiently validate the accuracy of the theory.

Through the experimental analysis, it is confirmed that optimal output voltage is achieved when both the vibration source and DVA are positioned at 1/2 of the tube length, and when comparing different spring constants, k = 20 provides the best output voltage. This is consistent with the damping results in the previous section and with our theoretical findings. Finally, Table 19 presents the calculation of the optimal power generation combination for exciting the first- and second-mode vibrations using Equation (67).

Despite the smaller output voltage of the large-magnet system compared to the small-magnet system, the calculation of the system’s output power, including the internal resistance, reveals that the small-magnet system outperforms the large-magnet system in both the first- and second-mode power outputs. Therefore, when both the shaker and DVA are placed at 1/2 of the tube length and using spring constant k = 20, the small-magnet system exhibits the optimal electrical power generation. Table 20 details the reduction in the experimental tube vibration amplitude, output voltage, and power for the first and second modes of the large- and small-magnet systems using different spring constants when the shaker and DVA are placed at 1/2 of the tube length. Through comparison, it can be observed that the small-magnet system demonstrates the optimal damping performance, while the large-magnet system exhibits superior output voltage. However, due to the larger internal resistance value of the large-magnet system, the small-magnet system achieves the optimal power generation.

## 5. Conclusions

This study designs an energy harvesting system that can be installed in industrial fluid pipelines, HVAC ducts, general engineering sewer systems, and subsea oil pipelines. The proposed model integrates a magneto-electric fluid–structural coupling system and provides better energy harvesting efficiency. First, a theoretical model is established, and then experiments are conducted to verify this model. This energy harvesting system collects the energy generated by the fluid flow using a DVA (dynamic vibration absorber) installed in the pipeline to absorb the vibrations, achieving vibration damping. Additionally, the repulsive force of the magnets installed in the DVA drives a PZT-equipped elastic steel in an MDDI VEH (vibration energy harvester) system, which has better voltage efficiency than the traditional VEH systems, paving the way for future applications in general pipeline facilities. The tasks completed in this study include the following:

1. Using Hamilton’s Principle to derive the equations of motion for a nonlinear fixed-free beam coupled with unsteady fluid flow and the DVA. The Biot–Savart Law is employed to develop an integrated model of this fluid–structure coupled DVA magnetic excitation MDDI energy harvesting system. The beam’s significant structural deformation and the tangential and normal forces on the pipe wall are considered as nonlinear fixed-free beams may experience large vibrations and deformations due to fluid excitation, which should be analyzed before executing vibration energy conversion.
2. Analyzing the system’s frequency response using the method of multiple scales (MOMS). The second part of the study involves determining the frequency response and amplitude of the system by exciting the flow tube’s vibration frequency to define the efficiency of this energy harvesting system. The integrated model of this fluid–structure coupled DVA magnetic excitation MDDI energy harvesting system is established and its electrical energy conversion efficiency is analyzed. The impact of different DVA parameters, including external excitation frequency, DVA mass, spring constant, and DVA position, on the system’s electrical energy conversion efficiency is examined as these are the key factors determining the system’s efficiency.
3. Conducting a simple experiment to verify the accuracy and feasibility of the theoretical model of this fluid–structure coupled DVA magnetic excitation energy harvesting system. The experiment involves fixing both ends of a flow tube at the water inlet and outlet, pressurizing the water flow with a variable-frequency pump at the inlet, and connecting the outlet to a storage tank via a conduit. The flow pipe is supported by a 3D-printed model. The experiment uses imc© to measure the damping effect and output voltage of the energy conversion for different DVA magnet masses and positions in the flow tube. The experimental and theoretical system output voltages for different DVA masses, positions, and spring constants are compared to verify the proposed model’s feasibility.

The following results were obtained from the experimental measurements and their comparison with the theoretical results:

1. When the DVA is installed at the vibration source, it significantly absorbs the vibration energy generated by the source, with the midpoint (1/2) of the tube performing better than the 1/4 position. Since the tube’s vibration energy is transmitted to the DVA, the tube experiences reduced vibration, while the DVA undergoes more intense vibrations.
2. The first-mode damping amplitude of the small-magnet system is about 18%, superior to the large-magnet system’s 12%. The second-mode damping amplitude is about 48% for the small-magnet system compared to 27% for the large-magnet system, indicating that the small-magnet system is more suitable as a damper for the flow tube.
3. For the current flow tube model, a spring constant of k = 20 is more suitable. Both the theoretical and experimental results show that the spring with k = 20 has significantly better damping effects across various modes compared to the springs with k = 13.3 and 12.5.
4. Measuring the internal resistance of the system reveals that the small-magnet system achieves the maximum power output when connected in series with a 50.45 K ohm resistor and the large-magnet system with a 95 K ohm resistor. Under these conditions, the small-magnet system has a higher power output than the large-magnet system, making it more suitable for use as a high-power damper.
5. The experiments changing three sets of springs and two different magnet masses show that larger magnets result in smaller spring vibrations regardless of the spring constant, while smaller magnets lead to more intense DVA vibrations. Thus, small magnets appear to be more capable of absorbing tube vibration amplitudes, leading to better damping effects and higher power output due to lower internal resistance, despite the larger magnetic repulsive force of large magnets causing greater elastic steel deformation and higher PZT output voltage.
6. The experimental results verify the feasibility of the proposed theoretical model. However, further research is needed to determine the optimal magnet size and spring constant for the best damping and power generation effects.

## Figures and Tables

**Table 1:** Maximum amplitude values of different modes excitation (dimensionless).

Excited Mode	1st Mode Amp.	2nd Mode Amp.	3rd Mode Amp.
1st mode	0.0878	5.8138 × 10^−12^	3.4677 × 10^−12^
2nd mode	4.9683 × 10^−13^	0.01725	3.2079 × 10^−16^
3rd mode	4.7497 × 10^−13^	2.5870 × 10^−14^	0.01694

**Table 2:** Comparison of amplitude of fixed-points plots and displacement in time responses for the flow tube (fluid–structure coupled beam) without additional damper.

	Fixed Points Plot	Time Response
1st mode	0.0878	0.0878
2nd mode	0.0173	0.0172
3rd mode	0.0169	0.0170

**Table 3:** Dimensionless amplitude of flow tubes (fluid–structure coupling beam) with and without the DVA attached.

	1st Mode	2nd Mode	3rd Mode
No TMD	0.0878	0.0172	0.0170
TMD (*x* = 0.25)	0.0825	0.0056	0.0034
TMD (*x* = 0.5)	0.0692	0.0043	0.0022

**Table 4:** Dimensionless displacement of the piezoelectric elastic beam caused by the DVA placed at different positions on the flow tube (fluid-structure coupling beam).

	1st Mode	2nd Mode	3rd Mode
TMD (*x* = 0.25)	0.0185	0.0009	0.0003
TMD (*x* = 0.5)	0.0222	0.0022	0.0004

**Table 5:** Dimensionless output voltage of the MDDI VEH with the damper placed at different positions.

	1st Mode	2nd Mode	3rd Mode
TMD (*x* = 0.25)	0.0769	0.0152	0.0127
TMD (*x* = 0.5)	0.0922	0.0378	0.0153

**Table 6:** Comparison of dimensional theoretical and experimental natural frequencies.

	1st Mode	2nd Mode	3rd Mode
Theo. (Hz)	12.886	30.652	57.471
Exp. (Hz)	12.210	29.291	56.641
Error (%)	5.246	4.444	1.444

**Table 7:** Voltage measurements for the small-magnet system with different load resistances.

Voltage (V)							
Ω	A	B	C	D	E	Average	Power (mW)
430	0.01341	0.01306	0.01313	0.01326	0.01340	0.01325	0.00041
10,000	0.28531	0.31143	0.31204	0.30489	0.30909	0.30455	0.00928
20,000	0.57152	0.57020	0.57633	0.57733	0.57935	0.57495	0.01653
30,000	0.78125	0.78260	0.77936	0.80679	0.79866	0.78973	0.02079
40,000	0.95133	0.94998	0.94172	0.97054	0.96278	0.95527	0.02281
50,000	1.08257	1.06119	1.06150	1.07731	1.09487	1.07549	0.02313
50,450	1.11676	1.11314	1.11645	1.11855	1.11524	1.11603	0.02469
51,000	1.08310	1.09361	1.08583	1.08459	1.08304	1.08603	0.02313
60,000	1.13522	1.14919	1.16478	1.16756	1.18949	1.16725	0.02271
70,000	1.23184	1.22401	1.23502	1.23554	1.25942	1.23717	0.02187
80,000	1.28294	1.27579	1.28429	1.28749	1.30656	1.28741	0.02072
Open-circuit voltage	1.58124	1.55870	1.56093	1.57052	1.57095	1.56847	

**Table 8:** Voltage measurements for the large-magnet system with different load resistances.

Voltage (V)							
Ω	A	B	C	D	E	Average	Power (mW)
430	0.00843	0.00849	0.00850	0.00842	0.00843	0.00846	0.00016
10,000	0.19845	0.19960	0.19994	0.19847	0.19962	0.19922	0.00397
20,000	0.38930	0.39306	0.39310	0.39345	0.38736	0.39125	0.00765
30,000	0.56633	0.56085	0.56699	0.56688	0.56765	0.56574	0.01067
40,000	0.72011	0.71781	0.72070	0.72212	0.72133	0.72022	0.01230
50,000	0.86169	0.86612	0.86223	0.86687	0.86335	0.86405	0.01493
60,000	0.98776	0.99144	0.99149	0.98860	0.98833	0.98952	0.01632
70,000	1.09244	1.08930	1.10459	1.09134	1.09356	1.09425	0.01711
80,000	1.18010	1.17803	1.18155	1.18086	1.17990	1.18009	0.01741
90,000	1.24568	1.25064	1.2575	1.26221	1.26023	1.25525	0.01751
92,000	1.27862	1.25892	1.27078	1.28101	1.28771	1.27541	0.01768
95,000	1.31192	1.30146	1.30254	1.30213	1.29894	1.30340	0.01788
98,000	1.30630	1.32030	1.32304	1.32119	1.31843	1.31785	0.01772
100,000	1.33391	1.33339	1.33102	1.32391	1.32874	1.33030	0.01770
110,000	1.38694	1.38147	1.38903	1.39027	1.38948	1.38744	0.01750
Open-circuit voltage	1.91610	1.89146	1.89380	1.88344	1.86312	1.88958	

**Table 9:** Comparison of dimensional theoretical and experimental displacement values of the tubes without DVA.

	1st Mode			2nd Mode		
	Shaker 1/4	Shaker 1/2	Shaker 3/4	Shaker 1/4	Shaker 1/2	Shaker 3/4
Theo. (mm)	1.59414	1.68435	1.59414	0.38618	0.40259	0.38618
Exp. (mm)	1.58068	1.68142	1.58507	0.37235	0.39631	0.39893
Error (%)	0.844	0.174	0.569	3.581	1.560	3.302

**Table 10:** Root mean square values of theoretical/experimental displacement for tubes with a large-magnet DVA placed at 1/2 of the tube length.

Theoretical/ Experimental	1st Mode			2nd Mode		
	Shaker 1/4	Shaker 1/2	Shaker 3/4	Shaker 1/4	Shaker 1/2	Shaker 3/4
Theo.Displ. (mm)	1.50635	1.46144	1.50635	0.28776	0.28587	0.28776
Redu.Amp. (mm)	0.08779	0.22291	0.08779	0.09842	0.11672	0.09842
%	5.507	13.234	5.507	25.486	28.992	25.486
Exp.Displ. (mm)	1.50979	1.46813	1.51705	0.29039	0.28972	0.30913
Redu.Amp. (mm)	0.07089	0.21329	0.06802	0.08196	0.10659	0.08980
%	4.485	12.685	4.291	22.012	26.896	22.510

**Table 11:** Root mean square values of theoretical/ experimental displacement for tubes with a small-magnet DVA placed at 1/2 of the tube length.

Theoretical/ Experimental	1st Mode			2nd Mode		
	Shaker 1/4	Shaker 1/2	Shaker 3/4	Shaker 1/4	Shaker 1/2	Shaker 3/4
Theo.Displ. (mm)	1.34984	1.36352	1.34984	0.21997	0.20084	0.21997
Redu.Amp. (mm)	0.24430	0.32083	0.24430	0.16621	0.20175	0.16621
%	15.325	19.048	15.325	43.040	50.113	43.040
Exp.Displ. (mm)	1.35158	1.37467	1.35949	0.23603	0.20660	0.22002
Redu.Amp. (mm)	0.22910	0.30675	0.22558	0.13632	0.18971	0.17891
%	14.494	18.244	14.232	36.611	47.869	44.847

**Table 12:** Root mean square values of theoretical/experimental displacement for tubes with a large-magnet DVA placed at 1/4 of the tube length.

Theoretical/ Experimental	1st Mode			2nd Mode		
	Shaker 1/4	Shaker 1/2	Shaker 3/4	Shaker 1/4	Shaker 1/2	Shaker 3/4
Theo.Displ. (mm)	1.48762	1.59968	1.53637	0.32963	0.38724	0.39292
Redu.Amp. (mm)	0.10652	0.08467	0.05777	0.05655	0.01535	0.00674
%	6.682	5.027	3.624	14.643	3.813	1.745
Exp.Displ. (mm)	1.49135	1.60842	1.54401	0.33059	0.39078	0.39544
Redu.Amp. (mm)	0.08933	0.07300	0.04106	0.04176	0.00553	0.00349
%	5.651	4.342	2.590	11.215	1.395	0.875

**Table 13:** Root mean square values of theoretical displacement/experimental displacement for tubes with a small-magnet DVA placed at 1/4 of the tube length.

Theoretical/ Experimental	1st Mode			2nd Mode		
	Shaker 1/4	Shaker 1/2	Shaker 3/4	Shaker 1/4	Shaker 1/2	Shaker 3/4
Theo.Displ. (mm)	1.47156	1.58327	1.50552	0.30944	0.36278	0.37949
Redu.Amp. (mm)	0.12258	0.10108	0.08862	0.07674	0.03981	0.00669
%	7.689	6.001	5.559	19.872	9.888	1.732
Exp.Displ. (mm)	1.47671	1.58718	1.51026	0.31087	0.36360	0.38196
Redu.Amp. (mm)	0.10397	0.09424	0.07481	0.06148	0.03271	0.01697
%	6.578	5.605	4.720	16.511	8.254	4.255

**Table 14:** Root mean square values of dimensional theoretical/experimental displacement for the large-magnet DVA system with different spring constants.

Theoretical/ Experimental	1st Mode			2nd Mode		
	k = 20	k = 13.3	k = 12.5	k = 20	k = 13.3	k = 12.5
Theo.Displ. (mm)	1.46144	1.49801	1.50527	0.28587	0.33996	0.35027
Redu.Amp. (mm)	0.22291	0.18634	0.17908	0.11672	0.06263	0.05232
%	13.234	11.063	10.632	28.992	15.557	12.996
Exp.Displ. (mm)	1.46813	1.50355	1.51061	0.28972	0.34182	0.35245
Redu.Amp. (mm)	0.21329	0.17787	0.17081	0.10659	0.05449	0.04386
%	12.685	10.579	10.159	26.896	13.749	11.067

**Table 15:** Root mean square values of dimensional theoretical/experimental displacement for the small-magnet DVA system with different spring constants.

Theoretical/ Experimental	1st Mode			2nd Mode		
	k = 20	k = 13.3	k = 12.5	k = 20	k = 13.3	k = 12.5
Theo.Displ. (mm)	1.36352	1.44757	1.45817	0.20084	0.27551	0.28839
Redu.Amp. (mm)	0.32083	0.23678	0.22618	0.20175	0.12708	0.11420
%	19.048	14.058	13.428	50.113	31.566	28.366
Exp.Displ. (mm)	1.37467	1.45469	1.46360	0.20660	0.27983	0.29278
Redu.Amp. (mm)	0.30675	0.22673	0.21782	0.18971	0.11648	0.10353
%	18.244	13.484	12.955	47.869	29.391	26.123

**Table 16:** Root mean square voltage of theoretical and experimental results for tubes with a large/small-magnet DVA placed at 1/2 of the tube length.

Large Magnet	1st Mode			2nd Mode		
	Shaker 1/4	Shaker 1/2	Shaker 3/4	Shaker 1/4	Shaker 1/2	Shaker 3/4
Theo. (V)	1.04574	1.35580	1.04574	0.01460	0.03681	0.01460
Exp. (V)	1.01995	1.30340	1.00110	0.01341	0.03381	0.01324
Error (%)	2.466	3.865	4.269	8.151	8.150	9.315
**Small Magnet**	**1st Mode**			**2nd Mode**		
	**Shaker 1/4**	**Shaker 1/2**	**Shaker 3/4**	**Shaker 1/4**	**Shaker 1/2**	**Shaker 3/4**
Theo. (V)	0.91366	1.12608	0.91366	0.03844	0.05065	0.03844
Exp. (V)	0.88341	1.08188	0.87720	0.03549	0.04642	0.03521
Error (%)	3.311	3.925	3.991	7.674	8.351	8.403

**Table 17:** Root mean square voltage of theoretical and experimental results for tubes with a large/small-magnet DVA placed at 1/4 of the tube length.

Large Magnet	1st Mode			2nd Mode		
	Shaker 1/4	Shaker 1/2	Shaker 3/4	Shaker 1/4	Shaker 1/2	Shaker 3/4
Theo. (V)	1.04574	0.80998	0.50129	0.01275	0.01037	0.00861
Exp. (V)	1.01007	0.78325	0.48673	0.01172	0.00957	0.00788
Error (%)	3.411	3.300	2.905	8.078	7.715	8.479
**Small Magnet**	**1st Mode**			**2nd Mode**		
	**Shaker 1/4**	**Shaker 1/2**	**Shaker 3/4**	**Shaker 1/4**	**Shaker 1/2**	**Shaker 3/4**
Theo. (V)	0.89235	0.79300	0.69102	0.02596	0.02066	0.01935
Exp. (V)	0.86239	0.76335	0.66700	0.02386	0.01910	0.01768
Error (%)	3.357	3.739	3.476	8.089	7.551	8.630

**Table 18:** Root mean square voltage of theoretical and experimental results for the large/small-magnet DVA systems with different spring constants.

Large Magnet (1/2)	1st Mode			2nd Mode		
	k = 20	k = 13.3	k = 12.5	k = 20	k = 13.3	k = 12.5
Theo. (V)	1.35580	0.98379	0.97550	0.03681	0.01560	0.01521
Exp. (V)	1.30340	0.94948	0.94088	0.03381	0.01428	0.01400
Error (%)	3.865	3.488	3.549	9.150	8.462	7.955
**Small Magnet (1/2)**	**1st Mode**			**2nd Mode**		
	**k = 20**	**k = 13.3**	**k = 12.5**	**k = 20**	**k = 13.3**	**k = 12.5**
Theo. (V)	1.12608	0.48588	0.24249	0.05065	0.02335	0.02302
Exp. (V)	1.08188	0.46942	0.23299	0.04642	0.02140	0.02111
Error (%)	3.925	3.387	3.918	8.351	8.351	8.297

**Table 19:** Comparison of RMS voltage and power between large- and small-magnet systems.

	1st Mode		2nd Mode	
	Large Magnet	Small Magnet	Large Magnet	Small Magnet
Theo. (V)	1.35580	1.12608	0.03681	0.050655
Power (mV)	0.01935	0.02513	0.000014	0.000051
Exp. (V)	1.30340	1.08188	0.03381	0.04642
Power (mV)	0.01788	0.02320	0.000012	0.000043

**Table 20:** Comparison table of optimal damping and power generation combinations.

Magnet Weight	45 g						35 g					
k	20		13.3		12.5		20		13.3		12.5	
Mode	1	2	1	2	1	2	1	2	1	2	1	2
Redu. Amp. (%)	12.685	26.896	10.579	13.749	10.159	11.067	18.244	47.869	13.484	29.391	12.955	26.123
Exp. (V)	1.3034	0.03381	0.94948	0.01428	0.94088	0.014	1.08188	0.04642	0.46942	0.0214	0.23299	0.02111
Power (mW)	0.01788	0.000012	0.00949	0.000002	0.00932	0.000002	0.0232	0.000043	0.00437	0.000009	0.00108	0.000009

## Data Availability

No new data were created.

## Appendix A. Dimensionless Parameters

$u=\frac{\bar{u}}{\bar{l}}$, $w=\frac{\bar{w}}{\bar{l}}$, $l=\frac{\bar{l}}{l}=1$, $v=\frac{\bar{v}}{\omega_{u}\bar{l}}$, $M=\frac{m_{f}}{m_{b}}$, $\tau =t\omega_{u}$, $\mu_{u}=\frac{{\bar{\mu }}_{u}}{(m_{b}+m_{f})\omega_{u}}$, $\mu_{w}=\frac{{\bar{\mu }}_{w}}{(m_{b}+m_{f})\omega_{w}}$, $\omega_{u}=\sqrt{\frac{EA_{b}}{(m_{b}+m_{f}){\bar{l}}^{2}}}$, $\omega_{w}=\sqrt{\frac{EA_{b}}{(m_{b}+m_{f}){\bar{l}}^{4}}}$, $\omega =\frac{\omega_{w}}{\omega_{u}}$, $I=\frac{I_{b}+I_{f}}{(m_{b}+m_{f}){\bar{l}}^{2}}$, ${\bar{k}}_{m}=\frac{k_{m}}{m_{b}{\omega_{M}}^{2}l}$, $m_{D}=\frac{m_{M}}{m_{b}}$, $\omega_{x}=\sqrt{\frac{{\bar{k}}_{m}}{m_{D}}}$.

## Appendix B. Coefficients Definitions

$B_{m}=\frac{\mu_{w}\int_{0}^{1}\phi_{n}\phi_{m}dx}{\int_{0}^{1}\phi_{n}\phi_{m}dx-\bar{I}(\int_{0}^{1}{\phi_{n}}^{\prime \prime }\phi_{m}dx)}$, $C_{m}=\frac{1}{2}\frac{\int_{0}^{1}{\phi_{n}}^{\prime \prime }\phi_{m}dx\int_{0}^{1}{\phi_{n}}^{\prime 2}dx}{\int_{0}^{1}\phi_{n}\phi_{m}dx-\bar{I}(\int_{0}^{1}{\phi_{n}}^{\prime \prime }\phi_{m}dx)}$, $D_{m}=\frac{Mv^{2}\int_{0}^{1}{\phi_{n}}^{\prime \prime }\phi_{m}dx}{\int_{0}^{1}\phi_{n}\phi_{m}dx-\bar{I}(\int_{0}^{1}{\phi_{n}}^{\prime \prime }\phi_{m}dx)}$, $E_{m}=\frac{\int_{0}^{1}\phi_{m}dx}{\int_{0}^{1}\phi_{n}\phi_{m}dx-\bar{I}(\int_{0}^{1}{\phi_{n}}^{\prime \prime }\phi_{m}dx)}$, $Q_{1}=\frac{\int_{0}^{1}\phi_{n}\phi_{m}dx}{\int_{0}^{1}\phi_{n}\phi_{m}dx-\bar{I}(\int_{0}^{1}{\phi_{n}}^{\prime \prime }\phi_{m}dx)}$, $Q_{2}=\frac{\int_{0}^{1}{\phi_{n}}^{iv}\phi_{m}dx}{\int_{0}^{1}\phi_{n}\phi_{m}dx-\bar{I}(\int_{0}^{1}{\phi_{n}}^{\prime \prime }\phi_{m}dx)}$, $Q_{3}=\frac{\int_{0}^{1}{\phi_{n}}^{\prime \prime }\phi_{m}dx\int_{0}^{1}{\phi_{n}}^{\prime 2}dx}{\int_{0}^{1}\phi_{n}\phi_{m}dx-\bar{I}(\int_{0}^{1}{\phi_{n}}^{\prime \prime }\phi_{m}dx)}$, $Q_{4}=\frac{\int_{0}^{1}{\phi_{n}}^{\prime \prime }\phi_{m}dx}{\int_{0}^{1}\phi_{n}\phi_{m}dx-\bar{I}(\int_{0}^{1}{\phi_{n}}^{\prime \prime }\phi_{m}dx)}$, $Q_{5}=\frac{\int_{0}^{1}{\phi_{n}}^{\prime \prime }\phi_{m}xdx}{\int_{0}^{1}\phi_{n}\phi_{m}dx-\bar{I}(\int_{0}^{1}{\phi_{n}}^{\prime \prime }\phi_{m}dx)}$, $Q_{6}=\frac{\int_{0}^{1}{\phi_{n}}^{\prime \prime }\phi_{m}dx\int_{0}^{1}{\phi_{n}}^{\prime 4}dx}{\int_{0}^{1}\phi_{n}\phi_{m}dx-\bar{I}(\int_{0}^{1}{\phi_{n}}^{\prime \prime }\phi_{m}dx)}$, $Q_{7}=\frac{\int_{0}^{1}{\phi_{n}}^{\prime \prime }\phi_{m}\int_{0}^{1}\int_{0}^{x}{\phi_{n}}^{\prime 2}dxdx}{\int_{0}^{1}\phi_{n}\phi_{m}dx-\bar{I}(\int_{0}^{1}{\phi_{n}}^{\prime \prime }\phi_{m}dx)}$, $Q_{8}=\frac{\int_{0}^{1}{\phi_{n}}^{\prime \prime }{\phi_{n}}^{\prime 2}\phi_{m}dx}{\int_{0}^{1}\phi_{n}\phi_{m}dx-\bar{I}(\int_{0}^{1}{\phi_{n}}^{\prime \prime }\phi_{m}dx)}$, $Q_{9}=\frac{\int_{0}^{1}{\phi_{n}}^{\prime \prime }{\phi_{n}}^{\prime 4}\phi_{m}dx}{\int_{0}^{1}\phi_{n}\phi_{m}dx-\bar{I}(\int_{0}^{1}{\phi_{n}}^{\prime \prime }\phi_{m}dx)}$, $Q_{10}=\frac{\int_{0}^{1}{\phi_{n}}^{\prime }\phi_{m}dx}{\int_{0}^{1}\phi_{n}\phi_{m}dx-\bar{I}(\int_{0}^{1}{\phi_{n}}^{\prime \prime }\phi_{m}dx)}$, $Q_{11}=\frac{\int_{0}^{1}{\phi_{n}}^{\prime 3}\phi_{m}dx}{\int_{0}^{1}\phi_{n}\phi_{m}dx-\bar{I}(\int_{0}^{1}{\phi_{n}}^{\prime \prime }\phi_{m}dx)}$, $Q_{12}=\frac{\int_{0}^{1}{\phi_{n}}^{\prime }{\phi_{n}}^{\prime \prime }\phi_{n}\phi_{m}dx}{\int_{0}^{1}\phi_{n}\phi_{m}dx-\bar{I}(\int_{0}^{1}{\phi_{n}}^{\prime \prime }\phi_{m}dx)}$, $Q_{13}=\frac{\int_{0}^{1}\phi_{m}dx}{\int_{0}^{1}\phi_{n}\phi_{m}dx-\bar{I}(\int_{0}^{1}{\phi_{n}}^{\prime \prime }\phi_{m}dx)}$, $Q_{14}=\frac{\int_{0}^{1}{\left.\phi_{n}\right|}_{x=L_{M}}\phi_{m}dx}{\int_{0}^{1}\phi_{n}\phi_{m}dx-\bar{I}(\int_{0}^{1}{\phi_{n}}^{\prime \prime }\phi_{m}dx)}$, $Q_{15}=\frac{1}{\int_{0}^{1}\phi_{n}\phi_{m}dx-\bar{I}(\int_{0}^{1}{\phi_{n}}^{\prime \prime }\phi_{m}dx)}$, $Q_{16}=\int_{0}^{1}{\left.\phi_{n}\right|}_{x=L_{M}}\phi_{m}dx$, $Q_{17}=\int_{0}^{1}\phi_{S}dx$, $Q_{18}=\int_{0}^{1}{\phi_{S}}^{iv}dx$, $Q_{19}=\int_{0}^{1}\phi_{S}\phi_{m}dx$, $Q_{20}=\int_{0}^{1}{\phi_{S}}^{\prime \prime 3}\phi_{m}dx$, $Q_{21}=\int_{0}^{1}{\phi_{S}}^{iv}{\phi_{S}}^{\prime \prime 2}\phi_{m}dx$, $Q_{22}=\int_{0}^{1}{\phi_{S}}^{\prime }{\phi_{S}}^{\prime \prime }{\phi_{S}}^{\prime \prime \prime }\phi_{m}dx$, $Q_{23}=\int_{0}^{1}{\phi_{S}}^{\prime \prime }\phi_{m}dx\int_{0}^{0.1}(\int_{0}^{\tau }(\int_{0}^{1}{\phi_{S}}^{\prime \prime }dx)e^{R\tau }d\tau )dx$, $Q_{24}=(\int_{0}^{1}\phi_{S}{\phi_{S}}^{\prime \prime }\int_{1}^{x}\int_{0}^{x}{\phi_{S}}^{\prime 2}dxdx+\int_{0}^{1}{\phi_{S}}^{\prime }\phi_{m}(\int_{1}^{x}\int_{0}^{x}{\phi_{S}}^{\prime 2}dxdx{)}^{\prime })$.
